# Genome-Wide Association Study for Traits Related to Plant and Grain Morphology, and Root Architecture in Temperate Rice Accessions

**DOI:** 10.1371/journal.pone.0155425

**Published:** 2016-05-26

**Authors:** Filippo Biscarini, Paolo Cozzi, Laura Casella, Paolo Riccardi, Alessandra Vattari, Gabriele Orasen, Rosaria Perrini, Gianni Tacconi, Alessandro Tondelli, Chiara Biselli, Luigi Cattivelli, Jennifer Spindel, Susan McCouch, Pamela Abbruscato, Giampiero Valé, Pietro Piffanelli, Raffaella Greco

**Affiliations:** 1 PTP Science Park, 26900 Lodi, Italy; 2 IBBA-CNR, Lodi, Italy; 3 CREA-Council for Agricultural Research and Economics, Rice Research Unit, 13100 Vercelli, Italy; 4 CREA-Council for Agricultural Research and Economics, Genomics Research Centre, 29017 Fiorenzuola d’Arda (Piacenza), Italy; 5 Department of Plant Breeding & Genetics, Cornell University, Ithaca, NY, United States of America; China National Rice Research Institute, CHINA

## Abstract

**Background:**

In this study we carried out a genome-wide association analysis for plant and grain morphology and root architecture in a unique panel of temperate rice accessions adapted to European pedo-climatic conditions. This is the first study to assess the association of selected phenotypic traits to specific genomic regions in the narrow genetic pool of temperate japonica. A set of 391 rice accessions were GBS-genotyped yielding—after data editing—57000 polymorphic and informative SNPS, among which 54% were in genic regions.

**Results:**

In total, 42 significant genotype-phenotype associations were detected: 21 for plant morphology traits, 11 for grain quality traits, 10 for root architecture traits. The FDR of detected associations ranged from 3 · 10^−7^ to 0.92 (median: 0.25). In most cases, the significant detected associations co-localised with QTLs and candidate genes controlling the phenotypic variation of single or multiple traits. The most significant associations were those for flag leaf width on chromosome 4 (*FDR* = 3 · 10^−7^) and for plant height on chromosome 6 (*FDR* = 0.011).

**Conclusions:**

We demonstrate the effectiveness and resolution of the developed platform for high-throughput phenotyping, genotyping and GWAS in detecting major QTLs for relevant traits in rice. We identified strong associations that may be used for selection in temperate irrigated rice breeding: e.g. associations for flag leaf width, plant height, root volume and length, grain length, grain width and their ratio. Our findings pave the way to successfully exploit the narrow genetic pool of European temperate rice and to pinpoint the most relevant genetic components contributing to the adaptability and high yield of this germplasm. The generated data could be of direct use in genomic-assisted breeding strategies.

## Introduction

Taxonomically, domesticated rice (*Oryza sativa*) can be subdivided into two subspecies -*O. sativa indica* and *O. sativa japonica*- and five major groups: indica, tropical japonica, temperate japonica, aus and aromatic [1]. Most accessions are cultivated in tropical humid areas; the recently derived group temperate japonica, adapted to temperate latitudes, however extend the area on which rice is grown, which currently amounts to over 164 MHa [2]. The temperate japonica group of *O. sativa* is mainly cultivated at temperate latitudes in the northern hemisphere (USA, southern Europe, north-western China and Japan) and accounts for ∼20% of total rice production [3]. Rice cultivation in temperate areas is therefore based mostly on temperate japonica accessions, but not exclusively: also some tropical japonica accessions are fruitfully grown outside of the tropics. Accessions adapted to temperate latitudes developed specific characteristics to cope with colder climates and the four-season cycle, such as resistance to lower temperatures and reduced photoperiod sensitivity [4, 5]. Rice breeding, as is the case for most other crop species, has long focused on increasing yield (52.4 per year in 1960–2010; [6]). More recently attention is beginning to be paid also to traits related to grain quality, like shape, texture and colour (e.g. [7]). Quality of rice grains is relevant especially for niche varieties like some Italian accessions used to make “risotto” [8]. Additionally, traits related to the efficiency of cultivation have become increasingly important (e.g. [9]): for instance, root traits may be linked to nutrient acquisition from the soil and can be used in breeding to reduce water requirements in irrigated rice production [10].

Plant architecture, grain and root morphology traits largely affect plant productivity and plant response to environmental stressors, and therefore represent targets for breeding schemes designed to increase both yield and quality of the final product [11–13]. The ideal plant architecture for rice was suggested as being characterized by a relatively small number of highly productive tillers [14, 15]; grain size is a major agronomic trait, associated to grain length, width, filling and thickness [16]. Root traits are involved in rice response to drought and in the uptake of nutrients from the soil [13].

During the last 10–15 years, the increasing availability of molecular markers (first SSRs —Short Sequence Repeats, now mainly SNPs— Single Nucleotide Polymorphisms) has allowed researcher and breeders to track segments of the genome linked to specific phenotypes of interest in QTL-mapping and genome-wide association studies (see [17] for a review). SSRs or SNPs mapped to the genome have thus been (and are) extensively used for Marker (or Gene) Assisted Selection (MAS, GAS) in plant breeding programmes [18]. MAS and GAS have been successfully employed also in rice breeding (reviewed in [19]), and molecular breeding applications bear the potential to help temperate rice contribute to the worldwide need for additional rice production in the next future (estimated 116 · 10^6^ tonnes by 2035; [20]). Results from QTL-mapping studies can also be used to improve the accuracy of genomic selection [21]. LD mapping based on genome-wide associations (GWAS), which exploits marker polymorphisms across all chromosomes [22], has become increasingly popular and powerful over the last few years and thanks to the emergence of more cost-effective, high-throughput genotyping platforms, has become a widely adopted approach for QTL mapping in plants [23]. Recently developed techniques based on the reduction of genome complexity, like Genotyping by sequencing (GBS) [24], are providing the marker density needed for GWAS, making the application of this procedure more feasible for different plant species.

In the present work, we report the results of a GWAS for 16 phenotypic traits related to plant morphology, grain quality (traits related to seed biometrics), and to the root apparatus, in a large collection of temperate japonica rice accessions genotyped for over 150000 SNPs. Previous GWAS works in rice have been published for a variety of traits (e.g. [25, 26]); genetic loci associated to plant architecture [14, 15], grain morphology [27] and root traits [13, 28, 29] have been identified. Most of the investigations on these agronomically relevant traits have been carried out on indica and tropical japonica groups, while very few studies are currently available in temperate japonica rice. Our study includes a wide range of shoot and root traits, employs a large set of SNPs and is focused mainly on temperate rice accessions. Detected polymorphisms may help reveal the biological processes underlying complex traits related to grain quality and other traits of agronomic importance, and could support rice breeding programmes in temperate areas.

## Materials and Methods

### Plant material

The accession panel used in this study included 391 *Oryza sativa* varieties from the Rice Germplasm Collection maintained at the CREA-Rice Research Unit (Vercelli, Italy). Varieties were selected based on the results of a genetic diversity analysis with 24 independent SSR markers [30]; the aim was to include the broadest possible range of genetically and phenotypically diverse temeperate rice accessions.

The sampled collection included 9 aromatic, 11 aus, 22 indica, 96 tropical japonica and 253 temperate japonica accessions. Most of these were temperate rice developed and selected in Italy (126 accessions), or developed elsewhere and adapted to Italian agro-climatic conditions (127 accessions). This collection was representative of the worldwide rice diversity [31], and was used to evaluate the population structure of the analysed rice accession panel.

The distribution of the 391 accessions per taxonomic group can be seen in Table 1. The complete list of the accessions used in this study, with information on taxonomic group and geographic origin is reported in S1 Table. All accessions were purified through single seed descent before genotyping and phenotypic evaluation. From all 391 accessions DNA was extracted for genotyping; phenotypes were collected on a subset of temperate and tropical japonica accessions: 153 for plant morphology and grain related traits, and 93 for root traits (see Table 1)

**Table 1:** Taxonomic classification of accessions.

Taxonomic group	Genotypes	Shoot/grain phenotyping	Root phenotyping
Aromatic	9	2	
Aus	11		
Indica	22	5	
Temperate Japonica	253	109	68
Tropical Japonica	96	37	25
*Total*	391	153	93

Number of accessions falling in the different taxonomic subgroups of *O. sativa*. Distribution for the 391 genotyped accessions, and for accessions phenotyped for plant morphology and grain related traits (“shoot” traits, 153 accessions), and for root traits (93 accessions).

### DNA isolation and genotyping

Total genomic DNA was isolated from three-week old leaves using the DNeasy Plant Mini Kit (QIAGEN) with a TECAN Freedom EVO150 liquid handling robot (TECAN Group Ltd, Switzerland). For each accession, a single individual plant was used. Whole-genome genotyping was carried out using Genotyping-By-Sequencing (GBS) technology [24]; DNA digestion was performed on 100 samples in 96-well plates using ApeKI, which was shown to cut every 1 kb on average in a *in-silico* digestion of the Nipponbare reference genome. Digested DNAs were ligated to 12 of 0.6 / adapter pairs (optimised to guarantee good quality libraries in rice), and the 96-plex library constructed according to the GBS protocol. The libraries were loaded into Genome Analyzer II (Illumina, Inc., San Diego, CA) for sequencing. Raw sequence data filtering, sequence alignment to the rice reference genome (*Os-Nipponbare-Reference-IRGSP-1.0*, [32]) and SNP calling from low-coverage GBS genotyping, were carried out using the *Tassel* GBS pipeline v3.0 provided by the Buckler Lab for Maize Genetics and Diversity [33, 34]. Default memory size parameters were modified according to data size, and a minimum of 5 tag counts from sequencing data was required. Sequences were aligned to the genome with the “Burrows-Wheeler Aligner” (BWA, [35]) using default parameters for genomes smaller than 2 G. Command lines and parameters used to generate the SNP dataset are reported in S2 Table.

A total of 166418 SNPs were called from GBS genotyping. Missing SNP genotypes were then imputed using the FILLIN (Fast, Inbred Line Library ImputatioN) algorithm in the Tassel GBS pipeline, accounting for complete homozygosity of domestic rice by considering rice accessions as fully inbred (inbreeding coefficient ≥ 0.99). FILLIN is based on haplotype reconstruction around recombination break points. Haplotypes are clustered per genotype similarity using the Hamming distance function. This information is eventually used to impute the target locus in an iterative approach that attemps, through a Markov Chain MonteCarlo (MCMC) process, to maximise the likelihood of the observed SNP calls given the unobserved imputed genotypes. When the FILLIN algorithm could not find haplotypes to satisfy any of the threshold requirements, the SNP locus was not imputed. After imputation, SNPs with call-rate ≥ 90% and MAF (minor allele frequency) > 1% (109888 SNPs) were used to investigate population structure and estimate linkage disequilibrium (LD). SNPs with call-rate ≥ 90% and MAF, respectively, ≥ 5% or ≥ 10% were used in the GWAS for plant and grain traits (57179 SNP) and for root (37827 SNP) traits. The distribution of SNP per chromosome is reported in Table 2.

**Table 2:** Distribution of SNPs per chromosome, before and after editing for call-rate and MAF.

Chromosome	SNP tot	SNP 153	SNP 93
OSA 1	19674	6894	4005
OSA 2	15332	5031	2695
OSA 3	14697	4228	1800
OSA 4	14344	4627	3510
OSA 5	11089	3587	2873
OSA 6	14245	4071	3257
OSA 7	12918	4836	3113
OSA 8	13201	5024	3260
OSA 9	9894	3448	2028
OSA 10	12603	5478	4356
OSA 11	16794	5984	3753
OSA 12	11627	3971	3177
*tot*	166418	57179	37827

Number of SNPs detected through GBS in the panel of 391 rice accessions: per chromosome distribution, before and after data editing for call-rate (> 0.90) and MAF (≥ 5% for plant and grain traits; > 10% for root traits)

### Population structure

The 391 rice accessions included in this study roughly cover the entire spectrum of domesticated rice populations, belonging to the 5 major phylogenetic groups traditionally identified (*Temperate japonica*, *Tropical japonica*, *Indica*, *Aromatic* and *Aus*) -or mixtures thereof. The underlying population structure was investigated using SNP genotype data to estimate the most likely number of clusters (*K*) into which the accessions can be grouped, and their degree of admixture. The following likelihood model was used to estimate the value of *K*:

$$\begin{array}{c} L\left(Q,F|g\right)=\sum_{i}\sum_{j}\left\{g_{ij}ln\left[\sum_{k}q_{ik}f_{kj}\right]+\left(2-g_{ij}\right)ln\left[\sum_{k}q_{ik}\left(1-f_{kj}\right)\right]\right\} \end{array}$$ (1)

where: *g*_*ij*_ is the observed number of copies (0 or 2, being 100% homozygous rice) of the reference allele at marker *j* in individual *i*; *q*_*ik*_ is the fraction of the genome of *i* contributed by population *k*; and *f*_*kj*_ is the reference allele frequency at marker *j* in population *k*. **Q** and **F** are the matrices of estimated *q*_*ik*_ and *f*_*kj*_, and have dimensions *I x K* (n. of individuals x n. of clusters) and *J x K* (n. of markers x n. of clusters) respectively; L(Q,F|g) is the log-likelihood of the matrixes **Q** and **F** given the observed genotypes (which is equal to the their joint probability density function: L(Q,F|g)=P(g|Q,F)).

The likelihood function in Eq 1 was maximised with a cross-validation procedure to identify the value of *K* that gives the best predictive ability [36].

The model was run for values of *K* varying from 2 to 18; a 5-fold cross validation scheme was adopted to estimate the prediction error for each value of *K*. Also the number of iterations needed to reach convergence (in the maximum likelihood estimation procedure) was monitored. The value of *K* that best fitted the data (the most likely number of clusters in the population) was determined based on the lowest prediction error and the smallest number of iterations for convergence. As described by Alexander et al. (2009, [36]), the number of iterations needed to make the solving algorithm converge rapidly increases when the data start to support poorly the tested number of clusters (*K*). From the matrix of contributions *Q* the probabilities of belonging to one of the clusters were derived, and accessions assigned accordingly.

An unweighted neighbor-joining (NJ) tree was constructed using a shared allele index based on a dissimilarity matrix estimated from the SNP dataset [37]. Based on seed availability, a subset of 153 rice accessions, predominantly temperate rice but still representative of the *Temperate* and *Tropical* japonica genetic diversity within the available rice collection, was selected and used for agronomic evaluations in field conditions, to record phenotypes related to plant morphology and grain quality. A further subset of 93 temperate and tropical japonica accessions was also evaluated for root architecture traits in controlled conditions. The list of the accessions selected for phenotyping -both plant and grain morphology and root traits- is reported in S1 Table. The genome-wide association study was therefore performed on the mentioned subsets of 153 and 93 accessions.

### Phenotype recording

Two experiments were conducted to record phenotypes on the rice accessions analysed: a field experiment for plant and grain morphology traits (153 accessions), and a greenhouse experiment for root traits (93 accessions). In the field experiment, rice plants were grown at the CREA-Rice Research Unit (Vercelli, Italy). Seeds were sown directly into dry soil in a randomized complete block design with three replicates. Each replicate (plot) measured 1.7 x 0.4 m and consisted of three rows, with 0.2 m inter-row and 0.3 m inter-plot spacing. Within each row, seeds were planted at 0.15 m from each other. A standard fertilization was applied, and conventional submersion was realised at 3°—4° leaf development stage up to one month before harvesting. At the post-emergence stage, two herbicide treatments were applied. During the growing season 7 agronomic traits related to plant and grain morphology were measured on five individual plants per replicate, following the specifications of the International Union for the Protection of New Varieties of Plants (IUPOV): plant height (PH), panicle length (PL), flag leaf length (FLL), flag leaf width (FLW), seed length (SL), seed width (SW) and seed length/width ratio (SR; see Table 3 for a complete list and description).

**Table 3:** Recorded traits.

Trait category	Trait name	Acronym	Trait description
Plant morphology (shoot)	Plant height	PH	Height of plant from soil surface to the tip of main panicle (inflorescence) at the time of maturity (cm)
	Panicle length	PL	Length of panicle (inflorescence) from the base (panicle neck) to the tip at the time of maturity (cm)
	Flag leaf length	FLL	Length of the flag leaf measured from leaf base to leaf tip at the time of maturity (cm)
	Flag leaf width	FLW	Width of the flag leaf measured at the widest portion of flag leaf lamina at the time of maturity (cm)
	Total shoot dry weight	SDW	
Grain morphology^1^	Seed length	SL	Length of the seed with hull (palea and lemma)^2^
	Seed width	SW	Width of the seed with hull (palea and lemma)
	Seed length/width ratio	SR	Ratio of seed length/ seed width (with hull)
Root morphology^3^	Root dry weight	RDW	
	Root length	RL	
	Root surface area	RSA	
	Root volume	RV	
	Number of tips	RT	
	Total length of thick roots	RL_TK	(diameter > 0.6 mm)
	Volume of thick roots	RV_TK	(diameter > 0.6 mm)
	Volume of vertical thick roots	RV_VTK	(diameter > 0.6 mm, root angle > 57°)

^1^Grain-morphology related traits were measured on rice seeds using the WinSeedle Pro V2007 software and STD4800 scanner (Regent Instruments Inc., Quebec, Canada).

^2^Seed = Mature spikelet filled with grain and surrounded by hull (palea and lemma)

^3^The root traits considered for GWAS were measured on different root angle sectors: RL, RV, RSA and RT on angle classes 3 and 4; RL_TK and RV_TK on any root angle but with root diameter > 0.6 mm; RV_VTK from angle class 4 and diameter > 0.6 mm.

For root phenotyping rice plants were grown under controlled conditions in plastic cylindrical mesh baskets (*Anelli s.r.l., Lodi, Italy*) placed at the top of PVC pipes (S1 Fig). PVC pipes were 60 high with a diameter of 14; mesh baskets were 5.4 cm high with a diameter of 7.2 cm and a mesh size of 2 mm. This size was large enough to allow root emergence from the basket without interference. Each PVC pipe was filled with field soil (silty clay loam, sieved to 5 mm), thoroughly mixed with 33% sand and inorganic fertilizer. The bottom of the pipe was covered with a non-woven fabric to allow free draining. PVC pipes were placed in a greenhouse based on a randomized block design with three replicates, for a total of 279 pipes. About 10 seeds for each of the 93 accessions were pre-germinated in petri-dishes for 48 h at 30°C. Afterwards, two seedlings per replicate were sown exactly in the centre of each basket at a depth of 1 cm. One week after emergence, the seedlings were thinned by selecting the healthiest ones. Plants were grown for six weeks at 28°C day / 24°C night at 75% relative humidity, under daylight conditions (16h light / 8h dark). Using a drip irrigation system, the pipes were supplied with 250 ml of water every day for the first 3 weeks and with 500 ml for the last three weeks. This amount of water —gauged in a previous test on a smaller subset of varieties (data not shown)— allowed to maintain aerobic conditions without unduly causing stress to the plants. From the third week, corresponding to the beginning of the tillering stage, 200 of inorganic fertilizer were supplied to each pipe once a week. Plant height (length from the soil to the tip of the longest leaf) was recorded on a weekly basis, as an indicator of shoot growth and plant health. At the end of the experiment (day 42), all shoots were cut at soil level, dried for three days at 80°C and weighted. Baskets were then gently extracted from the pipes and washed thoroughly to remove completely the soil from the roots. Roots were cut and classified based on their growth angle, measured by the position from where they emerged from the basket mesh. Four classes were thus defined with the following horizontal ground level angles: 0°-27° (1^st^ class), 27°-45° (2^nd^ class), 45°-57° (3^rd^ class), 57°-90° (4^th^ class). Roots were then placed on different glass trays according to the layer, taking care of spreading the smallest secondary roots, and scanned. Images were analysed with the WinRHIZO software (Regent Instruments Inc.). A total of 8 root traits were considered for the GWAS analysis: root dry weight (RDW), root length (RL), root volume (RV), root surface area (RSA), number of root tips (RT), length of thick roots (RL_TK), volume of thick roots (RV_TK) and volume of vertical thick roots (RV_VTK). RL, RV, RSA and RT were measured on roots from class 3 and 4; RL_TK and RV_TK were measured on roots of any angle but with diameter > 0.6 mm; RV_VTK on roots with diameter > 0.6 mm and angle class 4. During the root experiment, also shoot dry weight (SDW) was measured. All phenotypes were measured in 2012. Details are reported in Table 3.

### Estimation of linkage disequilibrium (LD)

The pairwise linkage disequilibrium (LD) among SNPs was estimated as *r*^2^:

$$\begin{array}{c} r^{2}\left(p_{A},p_{B},p_{AB}\right)=\frac{D^{2}={\left(p_{AB}-p_{a}p_{B}\right)}^{2}}{p_{A}\left(1-p_{A}\right)p_{B}\left(1-p_{B}\right)} \end{array}$$ (2)

where *p*_*A*_, *p*_*B*_ and *p*_*AB*_ are the frequencies of the AB halotype and of alleles A and B at the two SNP loci, and *D*^2^ = (*p*_*AB*_ − *p*_*A*_ *p*_*B*_)^2^ is the squared difference between observed and expected haplotype frequencies [38]. For LD estimation, all the 109888 SNPs with call-rate > 90% and MAF > 1% were used. Pairwise *r*^2^ values were averaged over SNPs grouped based on stepwise increasing base-pair distance (10 kbps steps: [0−10[, [10−20[, and so forth). The average LD as a function of base-pair distance was used to estimate LD decay in the rice accessions.

### Association study

Only SNPs with a call-rate ≥ 90% and minor alle frequency (MAF) ≥ 0.05 (plant and grain traits) or ≥ 0.1 (root traits) were used for the GWAS. Data filtering was applied separately to the two subsets of accessions phenotyped one for plant and grain morphology traits (153), and the other for root traits (93 accessions). This led to different numbers of SNPs used in GWAS: 57179 and 37827 for plant/grain and root traits respectively. The association between SNP genotypes and plant, grain and root phenotypes was tested fitting one SNP at a time in a mixed linear model of the following form (in matrix notation):

$$\begin{array}{c} y=1\mu +\text{Xb}+\text{Za}+e \end{array}$$ (3)

where **y** is the vector of phenotypic observations (averages across replicates); *μ* is the overall mean; **b** is the vector of SNP effects, with the corresponding matrix **X** of SNP genotypes (either 0 or 1 for the two homozygous classes —AA, BB); **a** is the vector of polygenic effects with the related incidence matrix **Z**; **e** is the vector of residuals. Polygenic and residual random effects were assumed to be normally distributed $a∼N(0,K\sigma_{a}^{2})$ and $e∼N(0,I\sigma_{e}^{2})$, where **K** is the kinship matrix among all accessions based on SNP genotypes and calculated according to Astle and Balding [39], **I** is the identity matrix, and $\sigma_{a}^{2}$ and $\sigma_{e}^{2}$ are the additive genetic and residual variances.

SNP effects estimated with model 3 were thus corrected for population relatedness, thereby reducing the risk of detecting spurious phenotype-genotype associations. For every SNP and trait, the null hypothesis of no association was tested with a Student *t*-test contrasting the model including the SNP effect with a reduced model including just the polygenic effect. The issue of testing multiple hypothesis (57,179 SNPs x 8 plant and grain traits, and 37827 SNPs x 8 root traits) was addressed by monitoring the false discovery rate (FDR, [40]) of detected associations.

### Software

SNP-calling, imputation of missing genotypes and the GWAS were carried out using the TASSEL pipeline and software package for genetic analysis [34, 41]. Population structure was inferred using the computer package *ADMIXTURE* [36]. Pairwise LD was estimated using the --r2 --ld-window-kb 5000 --ld-window 99999 --ld-window-r2 0 command in *Plink* [42]. Plots and data handling, preparation and processing, and NJ tree construction, were all performed with the open-source statistical programming environment *R* [43].

## Results and Discussion

### Genotypes and population structure: selecting material

**Fig 1:**
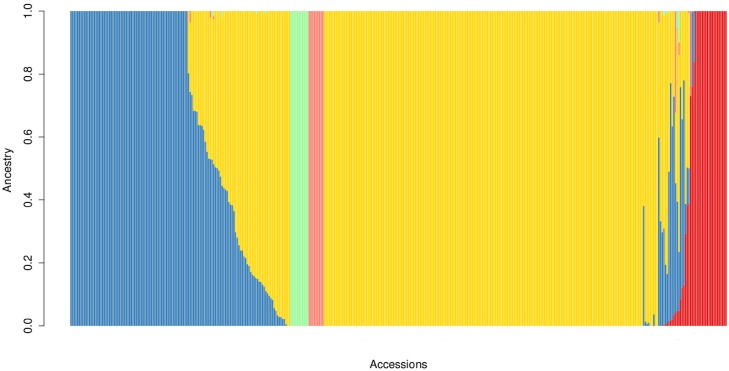
Stacked barplot for the ancestry of the available 391 rice accessions with K = 5.

**Fig 2:**
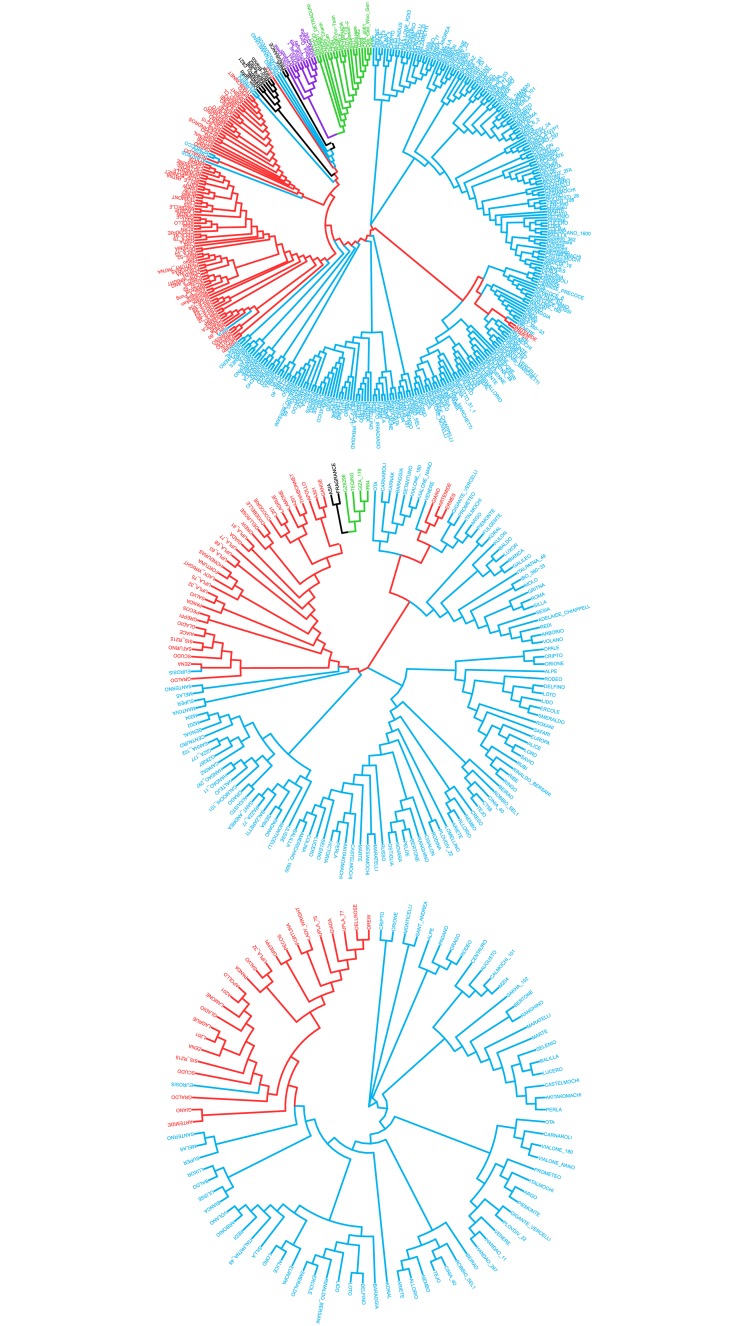
Neighbour-joining trees of the 391 accessions (left) and of the 153 (centre) and 93 (right) subsets used for plant & grain and root phenotyping respectively. Temperate japonica accessions in blue, tropical japonica in red, indica in green, aus in violet, and aromatic in black.

Whole-genome GBS genotyping of the 391 accessions in the European temperate rice diversity panel yielded a total of 166418 SNPs with an overall missing rate of 52%. GBS genotyping is indeed typically characterized by large rates of missing data (up to ∼50% on average [33]). After partial imputation with the *Tassel* GBS pipeline, the residual missing rate was 10.8%. Only SNPs with a call-rate ≥ 90% were used for downstream biostatistics analyses. For the association study, an additional filter on MAF was applied: MAF ≥ 0.05 for plant and grain traits (153 accessions), resulting in 57179 SNP and MAF ≥ 0.10 for root traits (93 accessions), resulting in 37827 SNP (see Table 2). Considering the largest panel of SNPs available (57179 SNPs), the average distance between SNPs was found to be 6.0 Kb, with 83.2% of the SNPs at the most 10 kb away from the closest neighbouring SNP locus. 54.0% (33634) of the identified SNPs was found within the sequence of annotated genes (MSU Osa1 Rice Genome Annotation [32]); of these, 60% (20302) were located in exons, 29% (9790) in introns, and the remaining 11% in apparently non-translated regions. A relevant fraction of the identified SNPs was therefore localized in transcribed regions, higher compared to previous reports where GBS markers in coding regions were observed with lower frequency, e.g. 39.5% in soybean [44], 46% in rice [29, 45] and 20.5% in oat [46]. SNP alleles were used to assess the population structure in the panel of accessions under examination. From the admixture analysis (Eq 1), the most likely value for the number of clusters (K value) was determined based on the cross-validation error in predicting to which group each accession belonged, and on the number of iterations to convergence. The prediction error reached a minimum for *K* = 5 and plateaued afterwards, while the number of iterations needed for the model to converge sharply increased for *K* > 6. The most likely number of clusters into which the analysed panel of accessions can be subdivided was, therefore, estimated to be *K* = 5 (Fig 1), which tallies with the number of main sub-populations distinguishable in *Oryza sativa* (indica, aus, aromatic, tropical and temperate japonica; [31]). However, the value of *K* = 6 has similar support from the admixture analysis (in terms of prediction error and iterations to converge). This is not unexpected, considering that some of the accessions are likely to be crosses between populations, and that the tropical japonica subgroup has been repeatedly reported to be constituted by two main clusters [25]. Considering the admixture plot for the 391 accessions with *K* = 5, 243 were assigned to temperate japonica, 85 to tropical japonica, 22 to indica, 9 to aromatic, 11 to the aus group, with probability higher than 0.6. Twenty-one accessions had a probability of assignment lower than 0.6, and appeared to be admixed, mainly between the temperate and tropical japonica groups (S3 Table). The unweighted NJ tree constructed to illustrate the phylogenetic structure of the rice panel (Fig 2) displayed the typical bipolar structure of *O. sativa* populations, with indica and aus accessions clearly separated from aromatic and japonica accessions [31, 47]. The NJ trees for the subsets of accessions used for plant and grain (153) and root (93) phenotyping showed that temperate rice accessions were purposely selected for GWAS, with the same relative proportion between the Tropical and Temperate japonica subgroups as in the whole panel.

Both in the admixture and NJ tree analyses, the admixed accessions most likely originate from interspecific breeding programmes, with extensive gene exchange between specific subpopulations. Within the japonica group, the temperate and tropical clusters were clearly distinguished, and represented most of the European accessions included in the study (65% and 24% of the panel, respectively). The analysis of population structure guided the selection of the most suitable set of accessions to be phenotyped in the field and root experiments. Mostly, temperate and tropical japonica accessions were selected, with indica and aromatic accessions as outgroups.

### Phenotypes and linkage disequilibrium: the basis for GWAS

On the selected set of informative temperate rice accessions, 5 plant morphology, 4 grain quality and 8 root traits were measured (Table 3). The analysis of the frequency distributions of the phenotypic classes indicates that all traits are quantitative and continuous (S2 Fig). The analysed phenotypic traits exhibited an overall broad variability, which is in principle suitable to be efficiently exploited in GWAS studies. All phenotypic traits were approximately normally distributed; a few distributions, though, were found to be slightly skewed (mainly towards lower values: e.g. SR, TRDW, RSA_4, RV_4, RL_4, RT_4), but none showed a multi-modal (e.g. bi-modal) pattern, or a clear separation in two or more classes (“discretization”).

**Fig 3:**
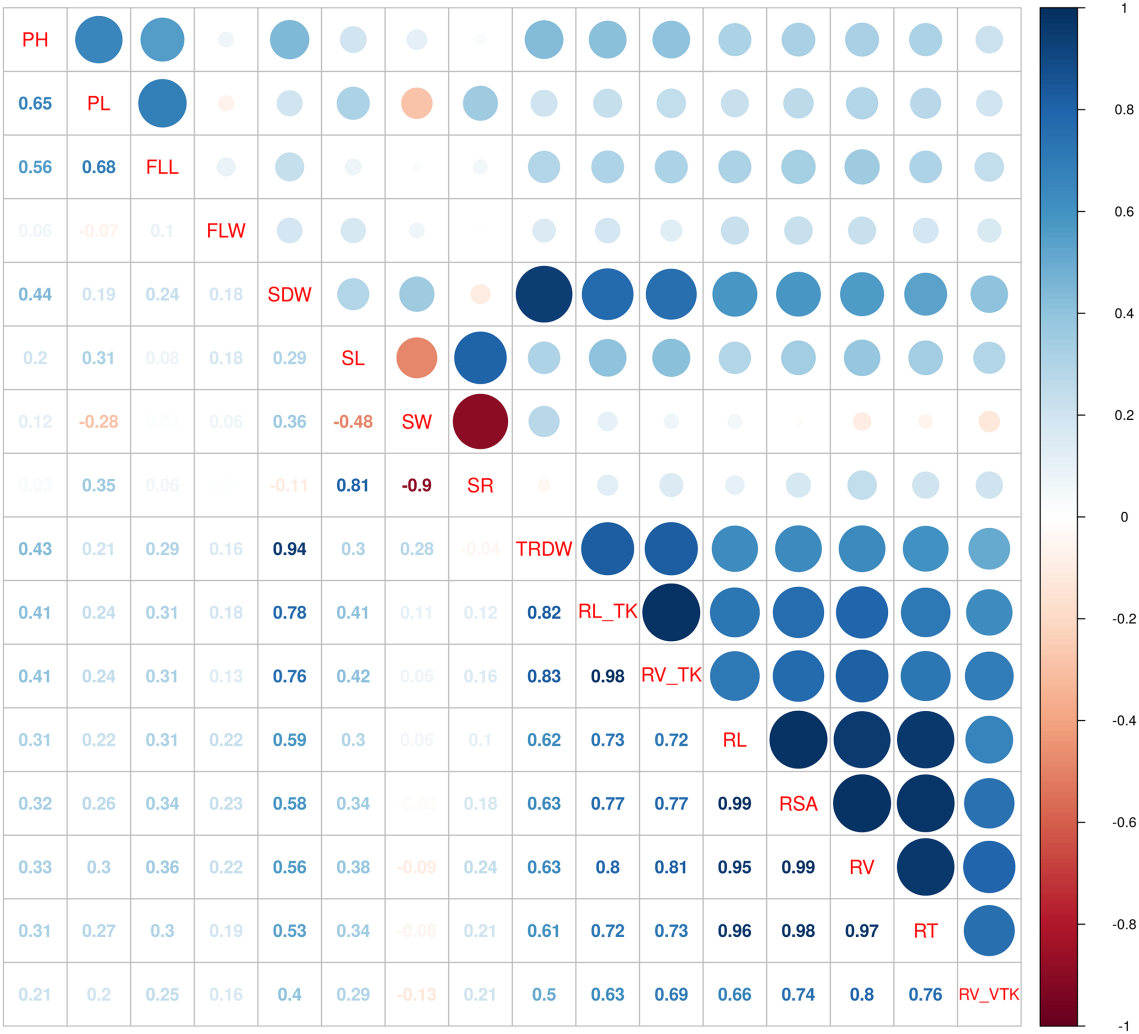
Phenotypic correlations among plant morphology, grain quality and root traits.

Most traits (14 out of 16) showed a larger than 2-fold difference between the minimum and the maximum values (Table 4). The coefficient of variation was on average 0.32 over all traits, and significantly larger for root traits ($\overset{-}{c.v.}=0.52$). Heritabilities were moderate-to-high, ranging from 0.58 for RV to 0.97 for FLW (Table 4), which is indicative of a good reproducibility of the experiments and a relevant genetic contribution to the observed variability in the measured traits. Phenotypic correlations appeared to cluster per trait group: grain quality, root morphology and, to a lesser extent, plant morphology (Fig 3). All correlations were positive, except for SL vs SW (*r*_*SL*,*SW*_ = −0.48), SW vs SL/SW ratio (*r*_*SW*,*SR*_ = −0.90) and PL vs SW (*r*_*PL*,*SW*_ = −0.28). The strongest positive correlations were *r*_*RL*,*RSA*_ = 0.99, *r*_*RSA*,*RV*_ = 0.99, *r*_*RL*_*TK*,*RV*_*TK*_ = 0.98, *r*_*RSA*,*RT*_ = 0.98, and *r*_*RV*,*RT*_ = 0.97. PH and PL showed a moderate positive correlation (*r*_*PH*,*PL*_ = 0.65), like PL and FLL (*r*_*PL*,*FLL*_ = 0.68). Shoot dry weight was positively correlated with all root traits; in particular, correlations with RDW, RL and RSA higher than 0.75 were observed.

**Table 4:** Descriptive statistics of collected phenotypes.

Trait category	Trait	Mean	SD	Max	Min	CV	*h*^2^
Plant morphology	PH (cm)	92.36	12.21	127.40	62.90	0.13	0.79
	PL (cm)	19.48	2.87	26.40	11.60	0.15	0.82
	FLL (mm)	243.28	52.56	380.00	135.00	0.22	0.86
	FLW (mm)	10.90	1.71	15.10	6.80	0.16	0.97
	SDW (g)	0.387	0.14	0.811	0.129	0.36	0.67
Grain quality	SL (mm)	9.17	0.98	11.75	7.19	0.11	0.89
	SW (mm)	3.36	0.50	4.42	2.43	0.15	0.80
	SR (ratio)	2.82	0.66	4.30	1.89	0.23	0.91
Root traits	RDW (g)	0.131	0.05	0.287	0.052	0.38	0.66
	RL (cm)	1916	962.6	4652	440.2	0.50	0.63
	RV (cm^3^)	0.805	0.416	1.933	0.206	0.52	0.58
	RSA (cm^2^)	136.90	69.66	301.20	33.71	0.51	0.60
	RT (#)	4283	2181.5	9343	837	0.51	0.64
	RL_TK (cm)	260.70	93.16	497.80	69.82	0.36	0.59
	RV_TK (cm^3^)	1.388	0.553	3.018	0.297	0.40	0.60
	RV_VTK (cm^3^)	0.1854	0.176	0.867	0.0016	0.95	0.67

**Fig 4:**
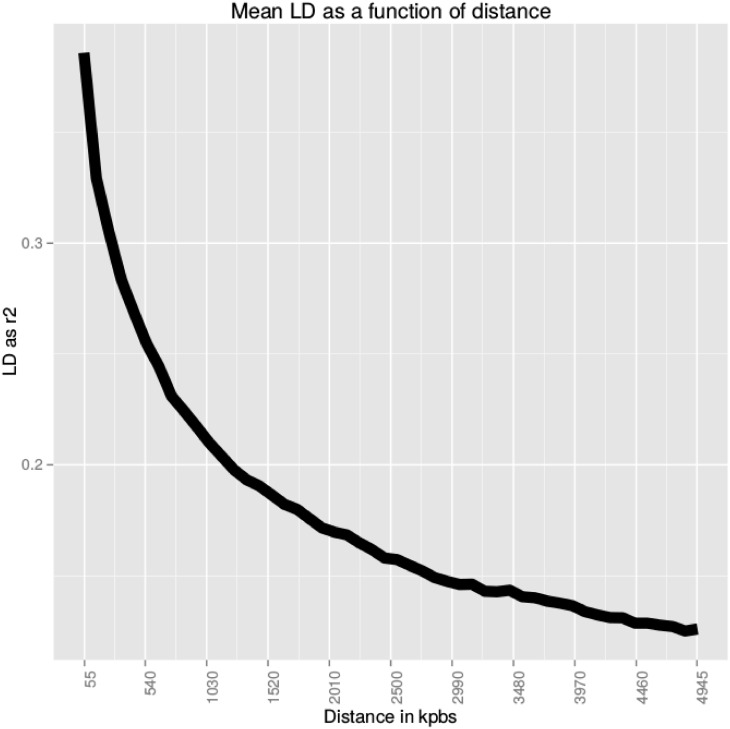
Average LD as a function of inter-marker distance estimated in the panel of 391 accessions.

Pairwise LD was estimated as *r*^2^ between all SNP markers genotypes in the 391 accessions included in the study. The average *r*^2^ as a function of inter-marker distance was used to estimate the LD decay in the population. Estimates of mean *r*^2^ at each 10 kbps incremental steps between 0 and 5000 kbps (5 Mbps correspond to ∼10 cM) indicated that average *r*^2^ starts at about 0.4 for very close markers (< 50 kbps), and decays to approximately 0.1 for SNPs as distant as 5 Mbps (Fig 4). The mean *r*^2^ drops below 0.2 beyond 1.25 Mbps inter-marker distance. Therefore, a considerable extent of LD was observed in the analysed rice population since *r*^2^ = 0.2 extends up to ∼1.25 Mbps (Fig 4). This value is higher compared to previous studies in domesticated rice, where LD as high as *r*^2^ = 0.2 was found from 100 kbps to maximum ∼500 kbps [29, 48]. These differences are likely to be due to the different experimental settings. First, the European temperate japonica accessions in our panel are highly related to each other, and the slow LD decay suggests that few historical recombination events occurred in this population. Secondly, SNP density can explain the higher LD estimated in our study: Courtois et al used 16664 markers (both SNPs and DArTs), Mather et al only 522. LD estimates tend to be higher with denser SNP panels (e.g. [49, 50]), and LD patterns tend to emerge clearly only at higher SNP densities (e.g. [51]). Data filtering criteria may also have played a role: both studies excluded markers with a MAF < 10%; MAF is known to influence LD estimates, though mainly in terms of *D*′, whereas *r*^2^ estimates appear to be robust to MAF (e.g. [52]). On the other hand, smaller sample sizes (Courtois et al. used 168 accessions, mainly Tropical japonica, Mather et al. 60 accessions equally spread over the Indica, Tropical and Temperate japonica groups) are known to bias upward LD estimates (e.g. [49]).

The wide phenotypic variability recorded in the analysed plant material and the long-range estimated LD, are both preliminary building blocks for the construction of a successful model for a genome-wide association study in temperate rice.

### GWAS: general aspects and specific associations

The traits included in the GWAS analysis were categorised in three classes: plant morphology, grain quality and root traits. The ample phenotypic variability and long-range LD extent, coupled with an average marker density of one SNP every 6 kbps, provided a good basis for whole-genome association mapping in temperate rice. The near normal distribution of most recorded phenotypes justified the use of a linear regression model for continuous traits in the association study (Eq 3), in which the population structure was properly accounted for through the kinship matrix.

#### Statistical significance of results

The FDR was estimated to monitor the effect of multiple testing: FDR for the associations in Table 5 varied from 3 · 10^−7^ to 0.92, with median 0.25. In GWAS studies it is however debatable whether multiple tests of a single global null hypothesis or individual tests of multiple hypothesis are concerned, and whether the threshold depends on the number of tests or on the prior probability of true SNP- phenotype associations [53]. In the latter case, the posterior odds of a true association would be given by its prior probability times the statistical power of the test ((*P*(reject *H*_0_|*H*_1_ is true)): probability of correctly rejecting the null hypothesis of no association when the SNP is truly associated to the phenotype), divided by estimated p-value:

$$\begin{array}{c} \frac{P(\text{true association})}{1-P(\text{true association})}=\frac{\text{prior probability }\cdot \text{ power}}{\text{p-value}} \end{array}$$

**Table 5:** Most significant associations between SNP genotypes and shoot, grain and root phenotypes in the analysed rice accessions.

	Trait	Marker	Chr	pos(kb)	p-value	FDR	Var(SNP)	interval(kb)	N.SNPs	QTL*	Candidate Gene
shoot	FLW	S1_4302318	1	4302	0.000076	0.101	0.0755		1	qFLW1	
	FLL	S1_28597986	1	28598	0.000013	0.307	0.1825	1.86	3		
	PH	S2_2826504	2	2827	0.000086	0.275	0.1113		1		LRK1 [61]
	FLL	S2_3104099	2	3104	0.000003	0.185	0.1586		1		
	FLL	S2_15780456	2	15780	0.000047	0.416	0.1061		1		
	PH	S2_29790001	2	29790	0.000051	0.241	0.1105		1	qPH-2 [58]	
	FLW	S3_12357940	3	12358	0.000035	0.065	0.0389		1		
	PL	S3_13264119	3	13264	0.000061	0.753	0.2457		1	qPLT3-2 [66]; pl3.1 [67]	
	FLW	S4_28827421	4	28827	0.000058	0.079	0.0423		1	Flw4 [54]	
	FLW	S4_31080152	4	31080	1.37 · 10^−11^	3.26 · 10^−7^	0.1914	775.7	36	qFLW4 [55]	LSCHL4 (NAL1); NarrowLeaf1)
	PL	S4_31520309	4	31520	0.000089	0.753	0.1198		1		
	FLW	S4_32193401	4	32193	0.000076	0.107	0.0383		1		
	PH	S5_17644414	5	17644	0.000009	0.0875	0.0947		1		
	FLL	S5_17644414	5	17644	0.000093	0.535	0.0554		1		
	PH	S6_22330734	6	22331	2.04 · 10^−7^	0.011	0.2392	278.55	11		D35 [62]
	PH	S6_23311513	6	23312	0.000100	0.275	0.1368		1		HDA702 [65]
	PH	S6_24644771	6	24645	0.000008	0.0851	0.1510	178.57	4	Qph6f [59]	
	PH	S7_16765878	7	16766	0.000021	0.148	0.0886		1		
	PL	S9_13253243	9	13253	0.000003	0.186	0.1882		1		
	FLL	S9_13253243	9	13253	0.000032	0.516	0.1487		1		
	FLL	S9_15078875	9	15079	0.000082	0.535	0.1229	50.1	2		
grain	SL	S1_42414831	1	42415	0.000066	0.766	0.0520		1		
	SL	S2_5453230	2	5453	0.000002	0.115	0.1415		1		
	SR	S2_5453230	2	5453	0.000098	0.252	0.0370		1		
	SR	S4_31243055	4	31243	0.000044	0.252	0.0299		1	gpp4	SLCHL4 (NAL1) [56]
	SW	S5_5401194	5	5401	0.000007	0.198	0.1201	254.23	13	qSW5 [77]	GW5 [71]
	SR	S5_5401194	5	5401	0.000083	0.252	0.0997	254.23	13	qSW5 [77]	GW5 [71]
	SR	S6_24916209	6	24916	0.000039	0.252	0.0201	55.57	3	qGL-6 [72]	
	SR	S7_18240854	7	18241	0.000082	0.252	0.0301		1		
	SR	S7_24575488	7	24575	0.000032	0.252	0.0962		1	grb7-2 [73]	
	SW	S7_24575488	7	24575	0.000063	0.252	0.0832		1	grb7-2 [73]	
	SR	S7_25119756	7	25120	0.000001	0.0530	0.1442	315.67	2	grb7-2 [73]	GE [70]
root	RV_TK	S1_1817023	1	1817	0.000010	0.511	0.0457	36.43	2		
	RV_TK	S2_23548832	2	23549	0.000065	0.927	0.0416		1	qRTT2-1 [78]	
	RL	S4_19970373	4	19970	0.000019	0.628	0.6834		1	qNOT4-2 [78]	
	RSA	S4_19970373	4	19970	0.000031	0.384	0.6934		1	qNOT4-2 [78]	
	RT	S4_19970373	4	19970	0.000035	0.542	0.6142		1	qNOT4-2 [78]	
	RV	S4_19970373	4	19970	0.000045	0.409	0.6959		1	qNOT4-2 [78]	
	RV_TK	S9_10237467	9	10237	0.000068	0.903	0.2890	79.87	9		
	RV	S10_15873490	10	15873	0.000082	0.173	0.0715	55.37	3	dt [79]	
	RV_TK	S10_16370558	10	16371	0.000039	0.302	0.3617	13.39	2	dt [79]	
	RV	S10_16370558	10	16371	0.000079	0.173	0.0672	13.39	2	dt [79]	

The SNP with the highest p-value is reported; FDR: false discovery rate; dt: drought tolerance;

*QTLs with no reference were obtained from the *Q-TARO* databases (http://qtaro.abr.affrc.go.jp/). The effect of the SNP is expressed in terms of proportion of the genetic variance explained by the SNP: $\hat{\sigma_{SNP}^{2}}={\hat{a}}^{2}\cdot p\left(1-p\right)$, where $\hat{a}$ is the estimated effect of the SNP, and *p* is the minor allele frequency (Zhang et al., 2010).

If 10 detectable signals are expected over the entire genome for any given trait, a plausible estimate of the prior odds of true association would be 10: 57179 = 1.75 · 10^−4^ for shoot and grain traits, and 10: 37827 = 2.64 · 10^−4^ for root traits. Assuming a power of 0.5, the posterior odds of the associations reported in Table 5 being true would range from a minimum of 0.88 (*P*(*x*) = 0.46) between SNP S6_23311513 and PH on chromosome 6, to a maximum of 6387094 (*P*(*x*) = 0.99) between SNP S4_31080152 and FLW on chromosome 4, with median value of 2.26 (*P*(*x*) = 0.69); 13 out of 42 associations (∼31%) had posterior odds > 4 (*P*(*x*) > 0.8).

These results indicate the value of looking at significance of GWAS results from multiple perspectives, and point to a high credibility of the associations detected in the analysed panel of temperate rice accessions.

#### Association mapping

**Fig 5:**
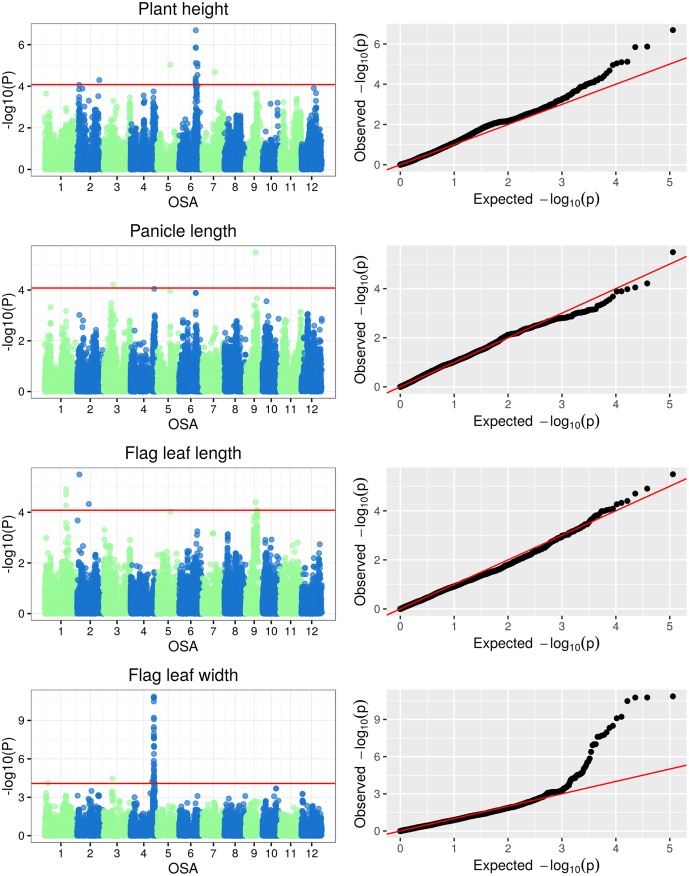
Manhattan and Q-Q plots of GWAS results for plant morphology traits.

**Fig 6:**
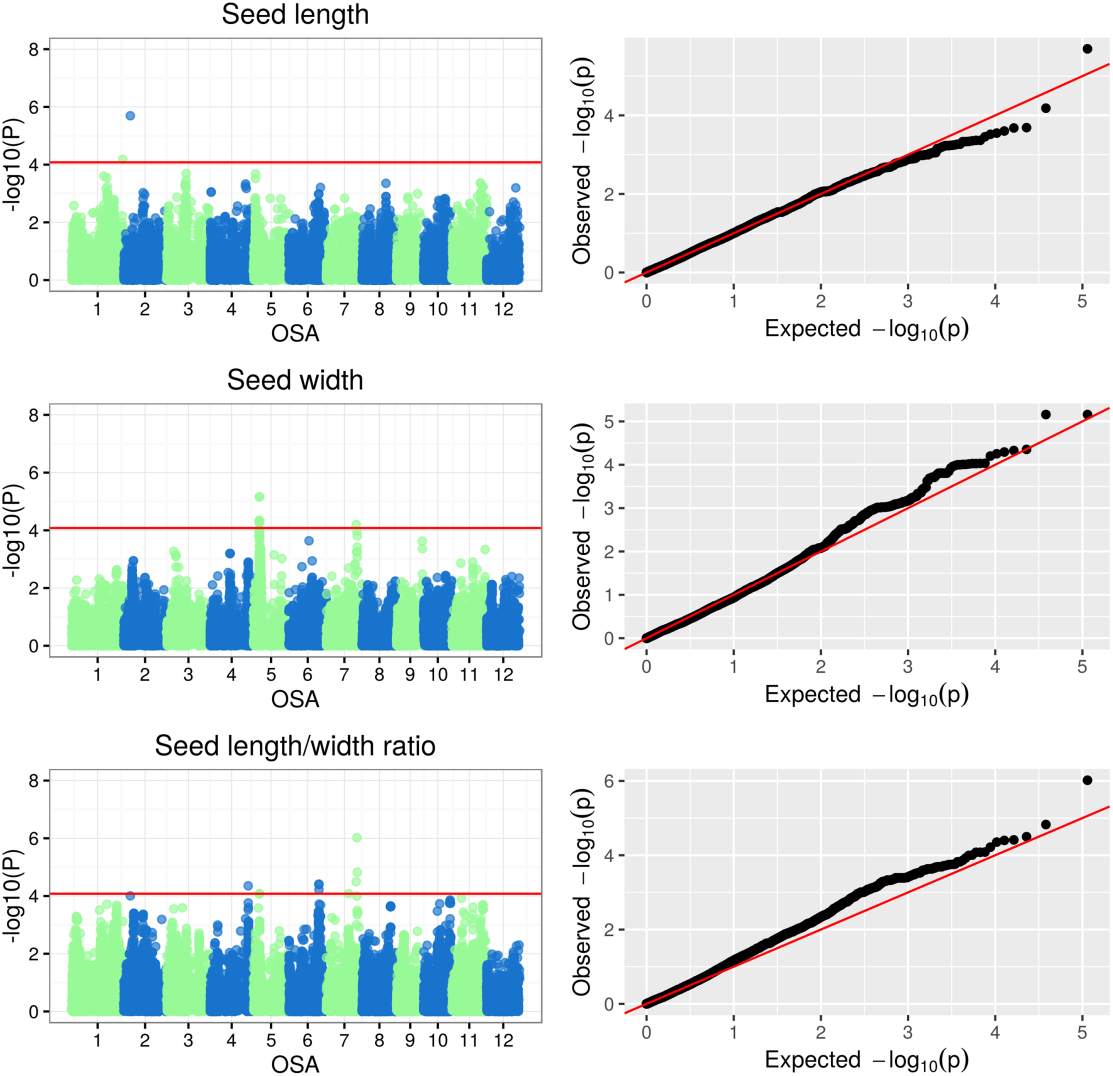
Manhattan and Q-Q plots of GWAS results for grain quality traits.

**Fig 7:**
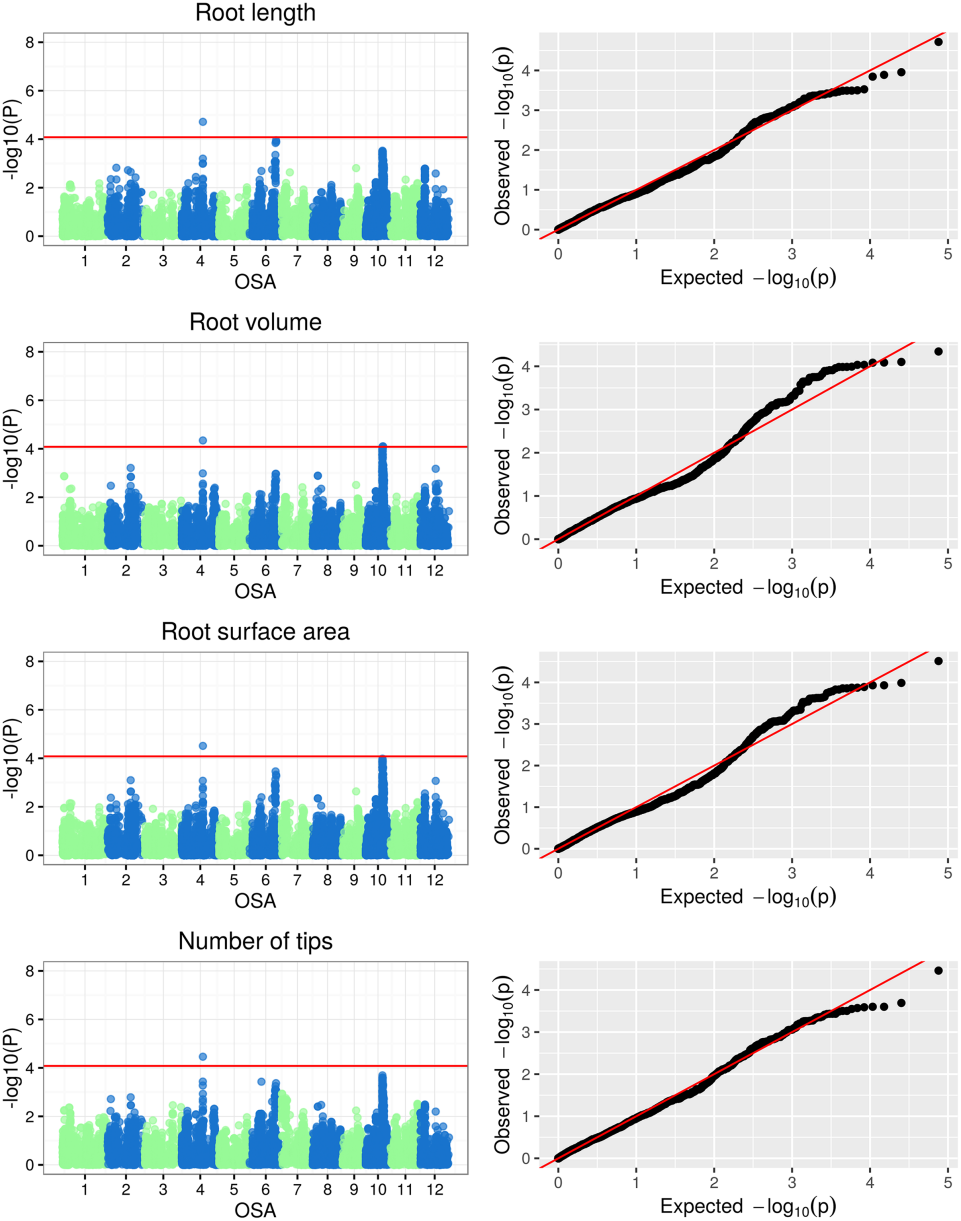
Manhattan and Q-Q plots of GWAS results for root traits.

**Fig 8:**
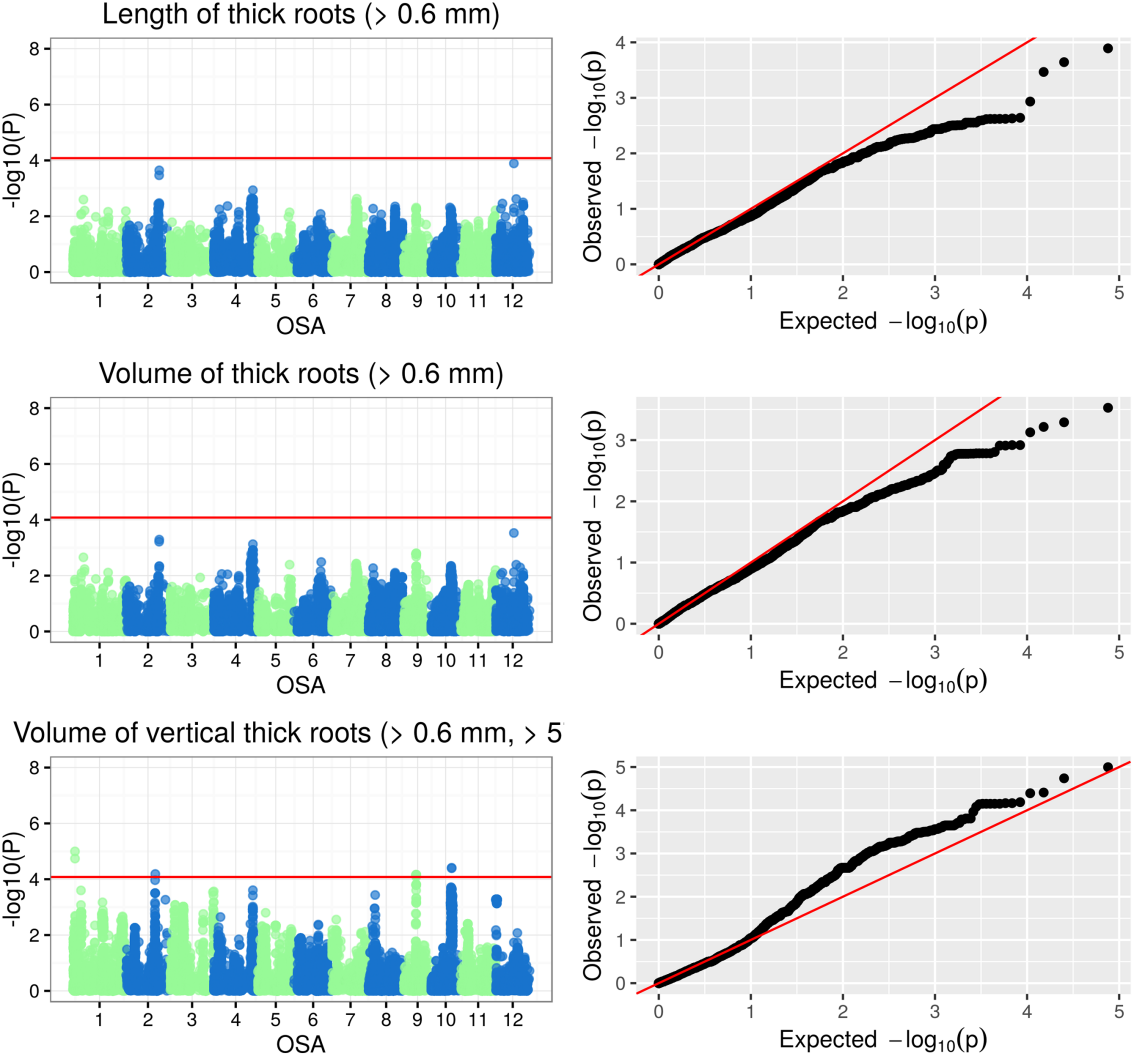
Manhattan and Q-Q plots of GWAS results for thick root traits.

**Fig 9:**
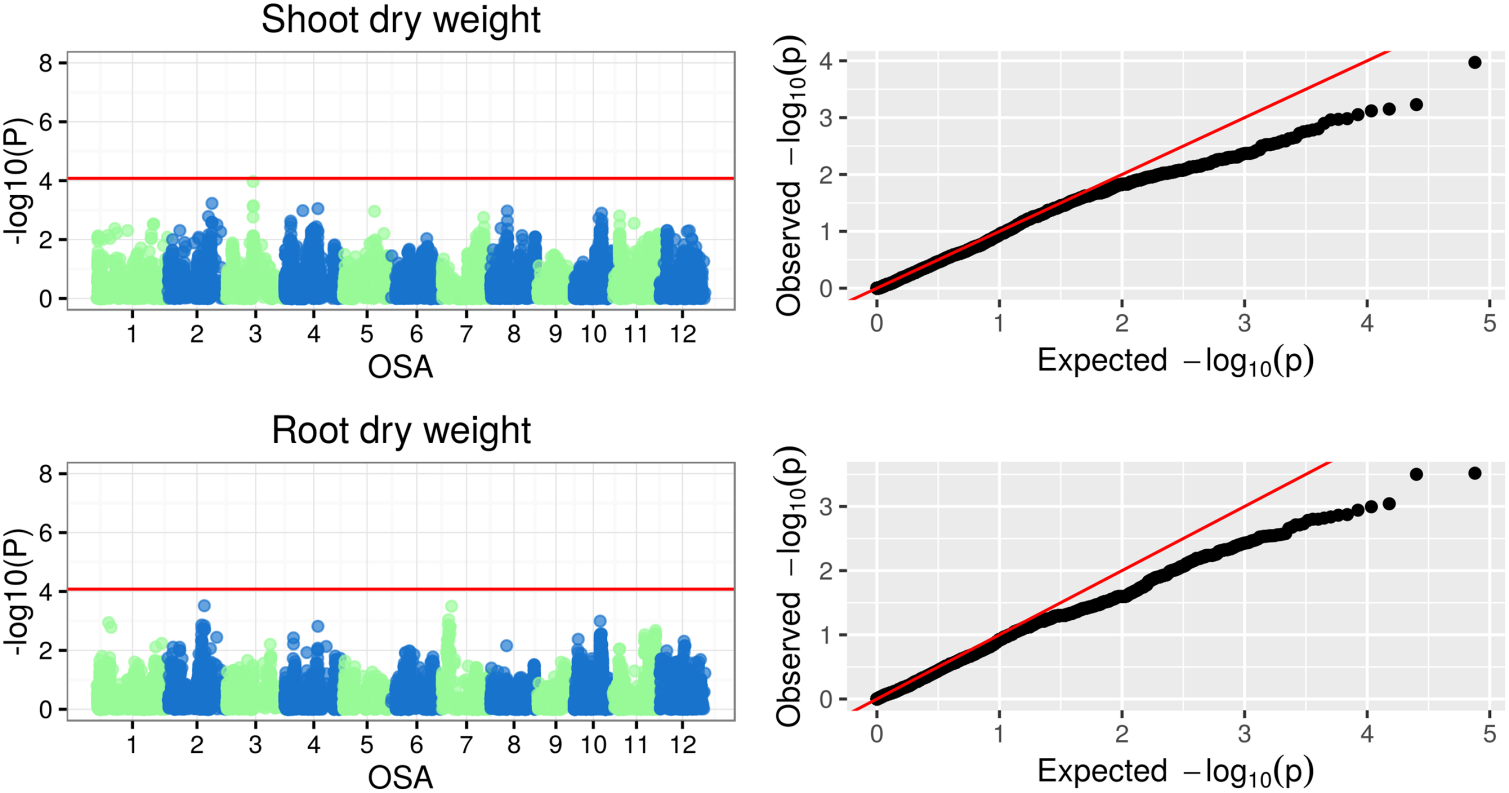
Manhattan and Q-Q plots of GWAS results for shoot and root dry weight.

The Manhattan plots of -log(p-values) and the Q-Q (quantile-quantile) plots of expected (under a Gaussian distribution) vs observed p-values for SNP-based genotype-phenotype associations for the three classes of plant traits under examination are reported in Figs 5, 6, 7 and 8. Additionally, the Manhattan and Q-Q plots for shoot and root dry weights are shown in Fig 9. The most significant associations (−*log*(*p*−*value*) > 4) detected in the GWAS study are listed in Table 5, where, for the largest peaks, only the most significant SNP is reported, together with the number of additional significant SNPs and the interval they span. The FDR for the associations in Table 5 ranged from 3.26 · 10^−7^ to 0.927 (average 0.32), with seven associations having a FDR < 0.1.

#### GWAS of plant morphology traits

A total of 21 significant associations (-log(p-value) > 4) between SNPs and the analyzed plant morphological traits were identified. The association with the lowest p-values were SNP S4_31080152 on OSA4 for FLW (p-value = 1.37 · 10^−11^), and SNP S6_22330734 on OSA6 for PH (p-value = 2.04 · 10^−7^). Other 4 associations for plant morphological traits had a p-value < 1 · 10^−5^ and included two SNP for PH (S5_17644414 and S6_24644771) and one each for FLL (S2_3104099) and PL (S9_13253243). Fourteen loci were associated with a morphological trait with p-value < 1 · 10^−4^ and one locus with p-value < 1 · 10^−3^ (Table 5). No significant associations were detected for SDW. In five cases, multiple SNPs belonging to the same chromosome region in full LD were identified as having the same level of significance for the same trait (3 SNPs on chromosome 1 for FLL, 36 SNPs on chromosome 4 for FLW, 11 SNPs on chromosome 6 at position 22331 kb for PH, 4 SNPs on chromosome 6 at position 24645 kb for PH and 2 SNPs on chromosome 9 for FLL). The involved genomic regions ranged from 1.86 kb to 775.7 kb. Some markers were significantly associated with more than one trait:marker S5_17644415 was found to be associated with PH and FLL, marker S9_13253243 with both PL and FLL.

Among the 21 loci significantly associated with plant morphologiy traits, three were in predicted genes for proteins with characterized functions, six co-localized with QTLs known to affect the corresponding traits, and three were located proximally to QTLs controlling plant morphology (Table 5). For FLW, three positional matches were observed on chromosome 4: SNP S4_28827421 was located in correspondence to the Flw4 QTL, mapped by Lim et al. [58] through whole genome re-sequencing of 178 rice F7 RILs (Recombinant Inbred Lines); SNP S4_31080152 and S4_32193401 co-localized with the QTL Qflw4, identified by Yue et al. [54] using a recombinant inbred population obtained from a cross between indica and japonica rice cultivars. For S4_31080152 a co-localization was found with the LSCHL4 gene, tightly linked to the Narrow leaf1 (NAL1) gene, which was reported in a narrow leaf rice mutant [55]. The NAL1 gene affects leaf width and plant height through its effects on cell division [57]. Its protein product is a plant specific protein known to be involved in cell cycle regulation by inhibiting cell division. Jiang et al. [57] demonstrated that NAL1 affects the expression of Auxin Responsive Factors (ARFs) required for the regulation of auxin response genes expression involved in auxin signalling. Moreover, this gene may act together with the YABBY family, implicated in leaf development, to regulate the formation of mid-ribs in leaves.

Two SNPs were found to be significantly associated to PH and co-localized with two known QTLs implicated in the control of plant height in rice: SNP S2_29790001 associated to qPH2.2, which was mapped on chromosome 2 using fifteen Chromosome Segment Substitution Lines [58]; and SNP S6_24644771 associated to Qph6f on chromosome 6, which was identified by Liang and co-workers (2011, [59]) using a population of 226 RILs. qPH2.2 has been postulated to be implicated in gibberellin (GA) biosynthesis as it has been located proximal to a putative GA-encoding gene [60]. SNP S2 2826504 co-localised with the candidate gene LRK1 encoding a Leucine-rich repeat receptor-like kinase (LRR-RLK); the LRK1 gene product was demonstrated to inhibit GA biosynthesis during internode elongation by down-regulating the expression of the ent-kaurene oxidase gene OsKO2 [61]. The PH-related markers S6_22330734 and S6_23311513, close to the Qph6f QTL, were associated to two different genes known to affect PH: *D35* and *HDA702*. The D35 gene, whose mutations causes a semi-dwarf phenotype, encodes the ent-kaurene oxidase enzyme, implicated in the gibberellin (GA) biosynthetic pathway [62]. It is well known that GAs modulate plant height by promoting cell elongation [63], thus mutations in D35 result in a reduction of GA biosynthesis and plant height. HDA702 corresponds to a histone deacetylase which increases growth and affects plant architecture through an epigenetic repression of OsNAC6, encoding for a transcription factor carrying a NAC domain implicated in the control of seedling root growth [64]. Moreover, down-regulation of rice HDA702 leads to the production of narrowed leaves and stems [65].

SNP S3_13264119 associated to PL co-localised with two QTLs on chromosome 3: qPLT3-2, identified using a Double Haploid (DH) population from an indica and japonica cross grown at nine different locations across Asia [66]; and pl3.1, mapped in an advanced backcross population derived from an *O. sativa* variety crossed with the wild relative O. rufipogon [67].

**Fig 10:**
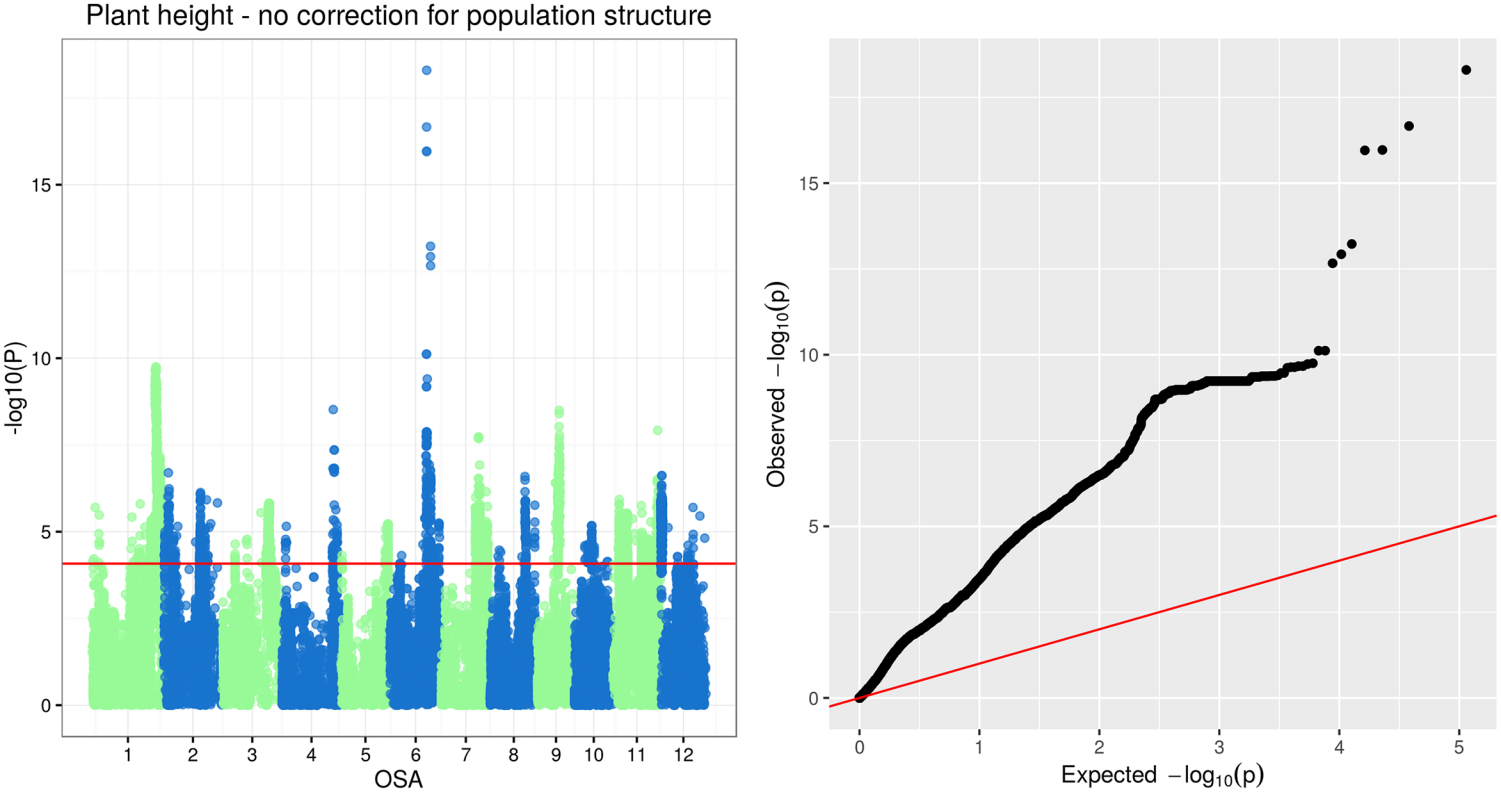
Manhattan and Q-Q plots of GWAS results for shoot and root dry weight.

The rice SD-1 semidwarfing gene is one of the most relevant genes employed in modern rice breeding, since the recessive allele results in shorter culms with improved lodging resistance and higher harvest index. The semi-dwarf phenotype was ascribed to a deficiency of active Gibberellins (GA) arising from a defective GA20-oxidase enzyme, a GA biosynthetic enzyme, which originates from a deletion of 280 bp within the coding region of Os20ox2 in indica rice, or from a substitution of a highly conserved Leu amino-acidic residue at position 266 in japonica rice ([68], a possible example of convergent selection). No SNP markers significantly associated with PH were identified in the region of sd-1 using the linear mixed model that included the kinship matrix. However, a strong association signal was detected for SNPs localized within and in proximity of the *sd-1* gene running a model that did not account for population structure (Fig 10 and S4 Table). Since the SD-1 gene is not common in upland rice varieties but it is conversely common in improved irrigated varieties, this result would imply that in the analysed panel of accessions the effects of population and polymorphisms in or close to the sd-1 gene are confounded. Apparently, in a set of temperate japonica accessions the sd-1 recessive allele linked to semi-dwarfism is still segregating, while in other accessions (e.g. the indica group) the same allele is either absent (wild type) or fixed (semi-dwarf lines).

#### GWAS of grain morphology traits

The association analysis for grain traits (Table 3) was also conducted on the same set of 153 informative rice accessions (Table 1; S1 Table). A total of 11 significant associations between SNPs and grain quality traits were identified. Three associations showed a p-value < 1 · 10^−5^: SNP S2_5453230 with SL, SNP S5_5401194 with SW and SNP S7_25119756 with SR. Eight SNP loci were associated with grain traits with p-value < 1 · 10^−4^ (Table 5). In three cases, multiple SNPs from the same genomic region were in full LD and had a comparable level of significance for the same trait (13 SNPs on chromosome 5 for SW and SR, 3 SNPs on chromosome 6 for SR and 2 SNPs on chromosome 7 for SR). The size of the associated genomic regions ranged from 36.43 kb to 254.23 kb. Similarly to morphological traits, also for grain traits specific SNPSs were significantly associated with multiple traits:SNP S2_5453230 was associated with SL and SR, SNP S5_5401194 and S7_24575488 were both associated with SW and SR (Table 5), indicating either pleiotropy or genetic linkage. Among the eleven loci associated with grain morphology, two were located in predicted genes for proteins with known functions (Table 5); qSW5/GW5 is a major QTL that negatively influences grain width and weight, by regulating cell proliferation during seed development. GW5 encodes for a 144-amino-acids uncharacterized protein that interacts with ubiquitin, suggesting that GW5 might be involved in the ubiquitin pathway, which has been reported to affect seed size in plants [69]. The SR-associated SNP S7_25119756 was located close to the cytochrome p450 gene GIANT EMBRYO (GE), implicated in the determination of embryo/endosperm size in developing rice seeds [70]. In addition, three loci co-localized with QTLs affecting the corresponding traits (Table 5): the aforementioned S5_5401194 was associated to the qSW5 QTL which corresponds to the gene GW5 [71]; the SR-related S6_24916209 was located in the qGL-6 region, shown to be involved in grain length determination by a sequencing-based high resolution genotyping mapping of rice RILs [72]; SNP S7_25119756, corresponding to the GE gene, co-localized also with the grb7-2 QTL, which affects grain breadth and was mapped using 209 RILs from a cross between Basmati rice and the contrasting breeding line Pusa 1121 [73]. This QTL was also located proximally to S7_24575488, associated to both SR and SW.

Recently, Chen and co-workers [74] characterized the GE gene, which encodes for a cytochrome P450 protein belonging to the CYP78 family, and controls the proper balance between embryo and endosperm tissues. This protein family represents a plant-specific class of cytochrome P450 mono-oxygenases, with distinct roles in plant growth and organ development, and is implicated in several biosynthetic pathways: e.g. phenylpropanoids, lipids, terpenoids, alkaloids and plant hormones biosynthesis [75]. GE influences endogenous indole acetic acid production in peroxisomes of young developing seeds cells and also regulates the expression of auxin-responsive and cyclin-related genes, thus influencing embryo development, cell elongation and cell cycle.

Altogether, several QTLs and candidate genes related to plant and grain morphology were demonstrated to significantly affect rice yield when transferred to a genetic background different from that where they were identified [11, 15, 76, 77].

#### GWAS of root traits

The analysis of the eight root traits (Table 3) was carried out on a set of 93 selected rice accessions representing temperate rice in the panel (Table 1; S1 Table). A total of 10 significant associations between SNPs and root traits were identified. All the associations showed p-values < 1 #x00B7; 10^−4^ (Table 5). In five cases, multiple markers belonging to the same genomic region were in full LD and showed comparable significance for the same trait: 2 SNPs on chromosome 1 for RV_TK, 9 SNPs on chromosome 9 for RV_TK, 3 SNPs on chromosome 10 for RV and 2 SNPs on chromosome 10 for RV_TK and RV. The length of the associated genomic regions ranged from 13.39 kb to 79.87 kb. Specific SNP markers were significantly associated with multiple root traits: SNP S4_19970373 on chromosome 4 was found to be associated with RL, RSA, RT and RV; SNP S10_16370558 on chromosome 10, associated with RV_TK and RV (Table 5).

Among the six SNPs significantly associated with root architecture, four co-localized with three QTLs known to influence root morphology (Table 5): S2_23548832 —associated to RV_TK— co-localised with qRTT2-1, a QTL controlling root thickness under two different developmental stages and during low moisture stress in a DH population derived from a cross between an indica and a japonica variety [78]; S4_19970373 —associated to RL, RSA, RT and RV— co-localised with qNOT4-2, a QTL that regulates the number of tillers under low moisture stress conditions in the same mapping population as qRTT2-1 [78]; S10_15873490 and S10_16370558 —associated with RV, and with RV_TK and RV, respectively— co-localised with a dt QTL (Gramene Accession Number = AQHP076), regulating root thickness during drought stress [79].

A detailed GWAS analysis of root traits in rice was recently conducted by Courtois et al [29] in a japonica rice panel. No common loci were identified between the two studies. The only positional match was observed between the q22 locus on chromosome 4 at 21386 kb in [29], associated to deep root biomass and number of roots > 30 deep, and S4_19970373 (at 19970 bps), associated to RL, RSA, RT and RV. The lack of obvious common findings is likely to be dependent upon the following: i) the germplasm panel under examination (the present panel was mainly composed of temperate japonica accessions while Courtois et al. focused their analysis on a panel tropical japonica accessions); ii) the experimental system (Courtois et al. based their phenotyping on 2D rhizoboxes, while our experiments were based on a 3D basket phenotyping); and iii) the average density of SNP markers (one marker per 22.5 kb in [29] and one marker every 11.1 kb in our GWAS analysis).

Root traits are gaining relevance in rice breeding for water-saving management systems. Currently, rice cultivation in temperate environments rely on large and consistent water supplies. Increasing concerns over climate change are urging a reduction of water for irrigation, which in turn calls for adaptability of rice varieties to reduced water availability. Root architecture plays a crucial role in adaptability of rice to such new circumstances, and introgression of QTLs and genes for root traits is demonstrated to increase rice yield under conditions of limited water availability (reviewed in [80]).

## Conclusions

A genome-wide association study was successfully carried out for a set of specific traits related to plant morphology, grain quality and root architecture in a unique panel of temperate rice accessions adapted to European temperate conditions. This is one of the largest studies on temperate rice for morphological and root traits, especially where the tridimensional architecture of the roots is considered. Although much is already known about root functioning in rice, there is still need for better understanding of root traits in the context of breeding strategies for growth under water-saving management systems.

The effectiveness of the presently developed platform for high throughput phenotyping, genotyping and association studies for temperate japonica was validated by confirming the involvement of specific QTLs and known genes in the control of plant, root and seed phenotypes of relevance for high yield and adaptation to the temperate climate (e.g. like the NarrowLeaf1 gene for flag leaf width on chromosome 4, and the qSW5 QTL for seed width on chromosome 5). A number of QTLs related to the different agronomically relevant traits have been detected. These results and the developed platform bear the potential of being used in breeding programmes to improve the quality of the grain and the drought tolerance of temperate rice accessions. The European GWAS platform was developed using a panel constituted mostly of adapted temperate japonica breeding lines rather than on a diversity panel providing the opportunity to apply the obtained association results directly to ongoing and future breeding programs in Europe and other temperate rice-growing areas (e.g. Japan, northern China, USA). The identified segregating haplotypes are valuable tools to define the most suitable parents for crossing, to enhance the frequency with which these haplotypes appear in the progenies.

Marker Assisted Selection for specific favorable haplotypes will enable us to increase breeding efficiency and decrease cost by reducing the number of plants to be brought to the next generation of breeding or that need to be in-depth phenotyped.

## Supporting Information

S1 Fig Root phenotyping experiment. Root phenotyping experiment. A) The various phases of the experiment. B) The four layers defined according to the root growth angle.
(PDF)

S2 Fig Trait distributions. The phenotypic distribution of the plant morphology, grain quality and root traits used for the genome-wide association study.
(PDF)

S1 Table Rice accessions. List of accessions used in the study with their geographical origin, commercial class, taxonomical group and collected pheno- types (153 accessions phenotyped for plant and grain morphology; a subset of 93 accessions phenotyped also for root traits).
(PDF)

S2 Table Command lines. Command lines and parameters used to generate the imputed non-filtered SNP dataset with Tassel GBS pipeline 3.0. Command lines with the “Plugin” suffix are part of the TASSEL pipeline; filenames and directories are not listed. In order to run the “FastImputationBitFixedWindowPlugin”, a tab-separated file with an inbreeding coefficient of 0.99 for each taxon was provided. When not reported, default parameters were used.
(PDF)

S3 Table Probability of assignment. List of accessions used in the study with the taxonomical group they have been assigned to and the probability of assignment to each of the 5 major groups based on their SNP genotypes.
(PDF)

S4 Table SD1 without correcting for population structure. Associations between SNP genotypes and plant height in the region of the gene SD1 on chromosome 1, from a linear GWAS model without accounting for population structure.
(PDF)
