# Supplementary material for: Genome-Wide Association Study for Traits Related to Plant and Grain Morphology, and Root Architecture in Temperate Rice Accessions
Source: PLoS One. 2016 May 26;11(5):e0155425. doi: 10.1371/journal.pone.0155425 (PMC4881974; doi:10.1371/journal.pone.0155425)
Supplement: S1 Fig — Root phenotyping experiment. A) The various phases of the experiment. B) The four layers defined according to the root growth angle. (PDF) [file pone.0155425.s001.pdf]

Genome-wide association study for traits related to plant and grain morphology, and root architecture in temperate rice accessions

Filippo Biscarini[1,*] et al.
1 Department of Bioinformatics and Biostatistics, PTP Science Park, Lodi, Italy
∗ **E-mail: filippo.biscarini@ptp.it**

Root phenotyping experiment. A) The various phases of the experiment. B) The four layers defined according to the root growth angle.

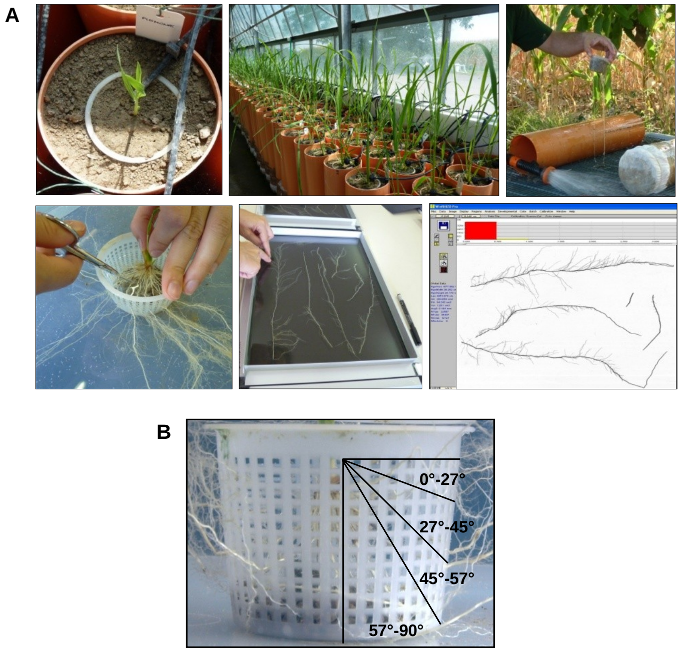

Supplementary Figure 1. Root phenotyping experiment. A) The various phases of the experiment. B) The four layers defined according to the root growth angle.
