# Supplementary material for: Genome-Wide Association Study for Traits Related to Plant and Grain Morphology, and Root Architecture in Temperate Rice Accessions
Source: PLoS One. 2016 May 26;11(5):e0155425. doi: 10.1371/journal.pone.0155425 (PMC4881974; doi:10.1371/journal.pone.0155425)
Supplement: S2 Fig — The phenotypic distribution of the plant morphology, grain quality and root traits used for the genome-wide association study. (PDF) [file pone.0155425.s002.pdf]

Genome-wide association study for traits related to plant and grain morphology, and root architecture in temperate rice accessions

Filippo Biscarini[1,*] et al.
1 Department of Bioinformatics and Biostatistics, PTP Science Park, Lodi, Italy
$*$ **E-mail: filippo.biscarini@ptp.it**

The phenotypic distribution of plant morphology, grain quality and root traits are shown in figs. 1 to 3

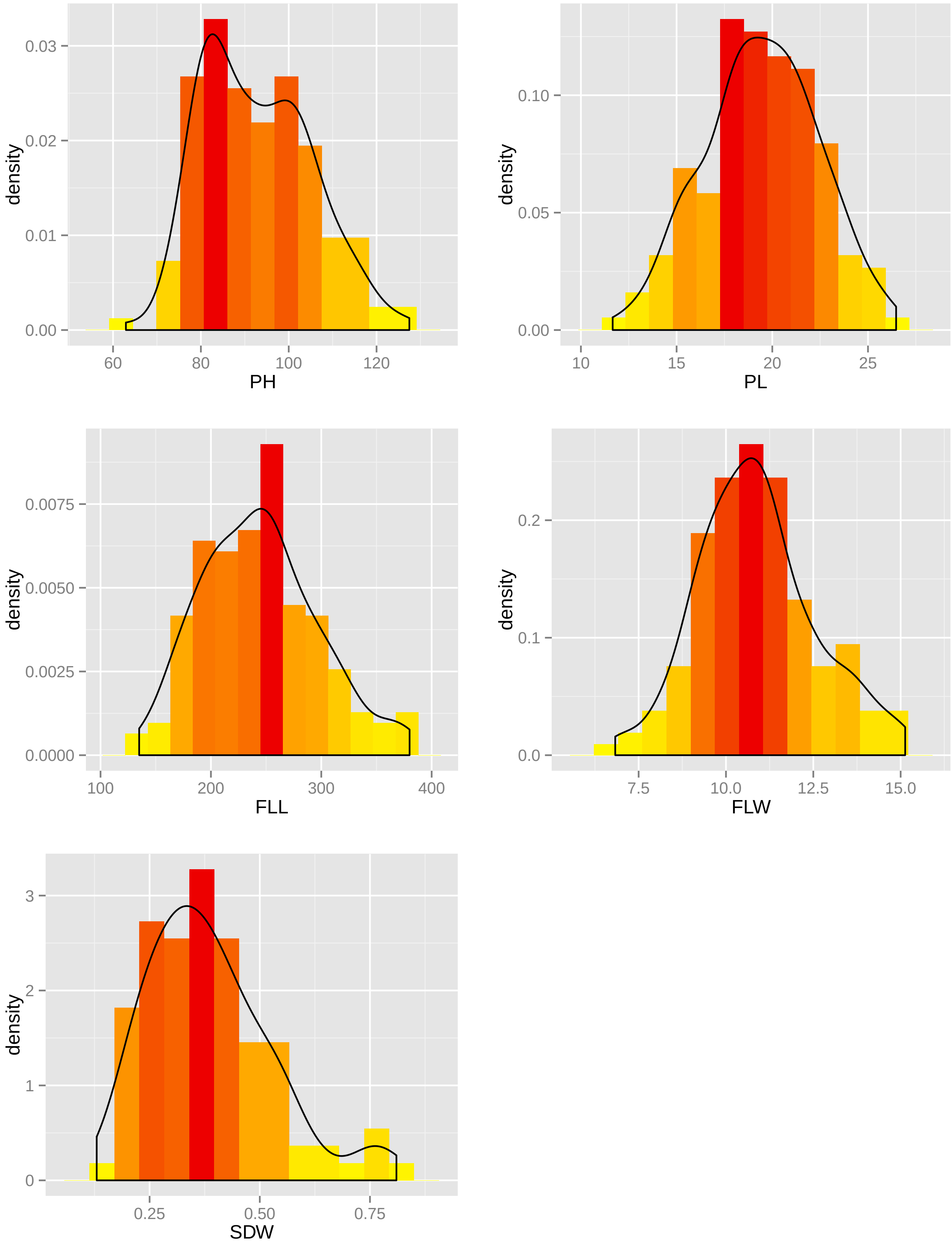

Figure 1: Phenotypic distribution of plant morphology traits

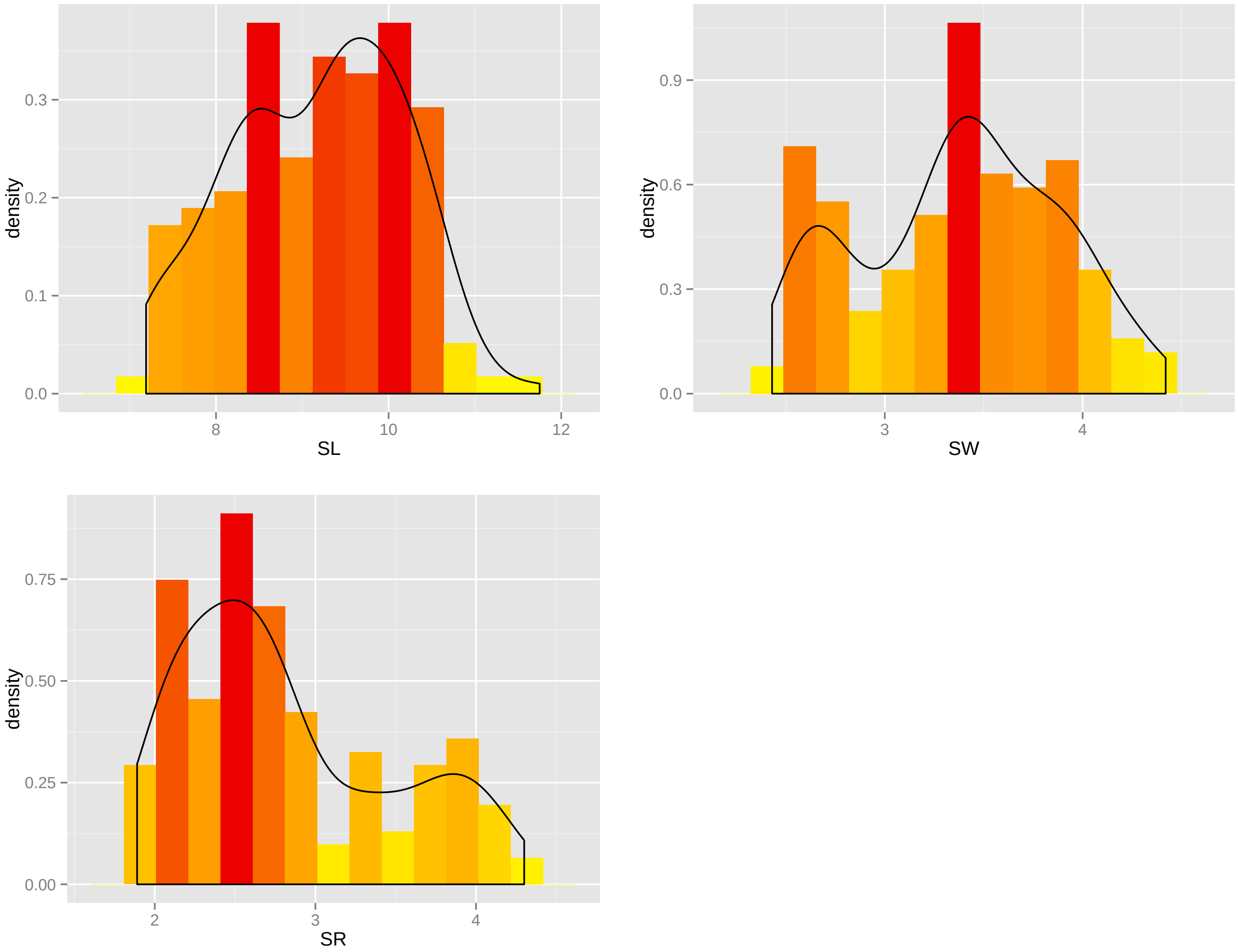

Figure 2: Phenotypic distribution of grain quality traits

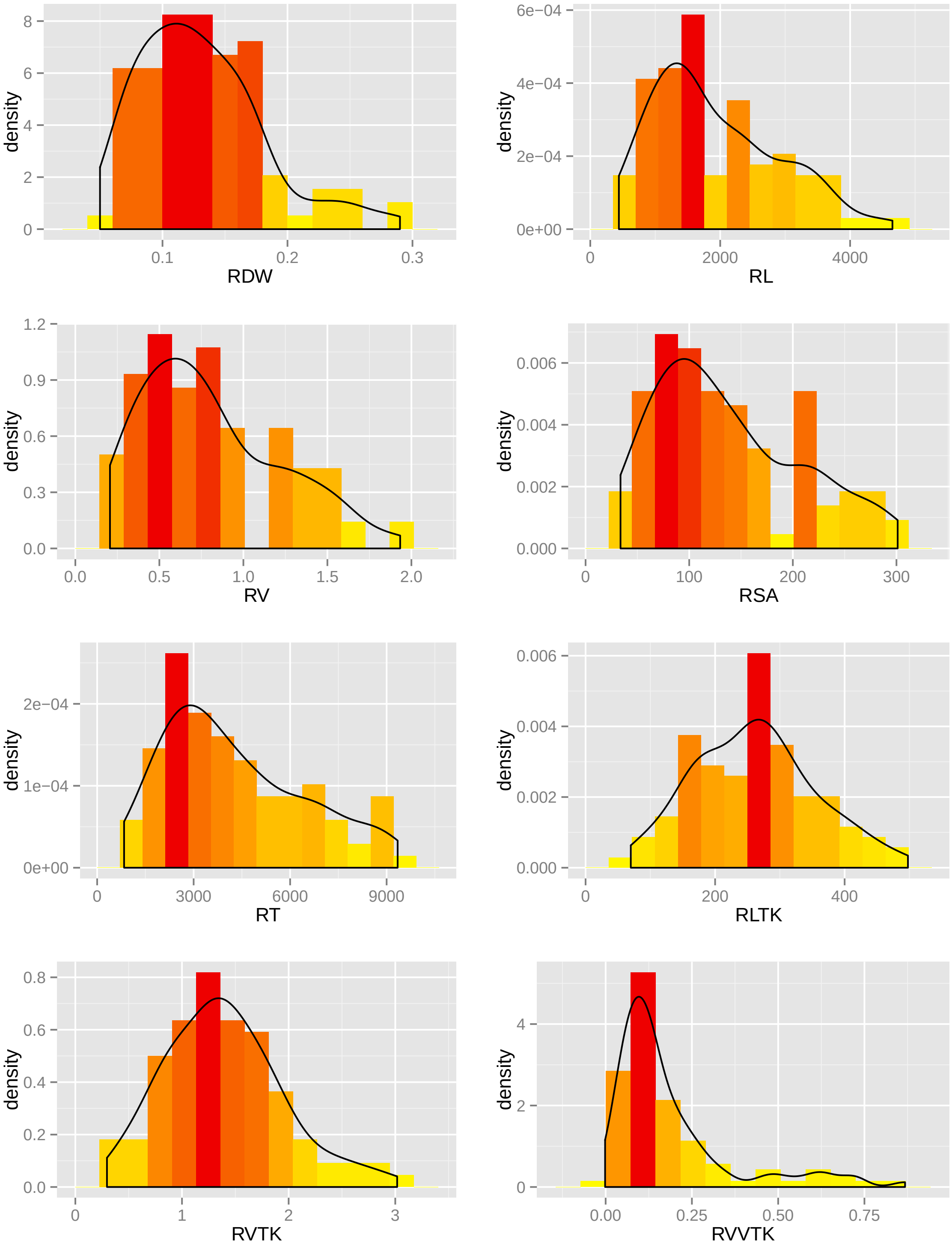

Figure 3: Phenotypic distribution of root traits
