# Supplementary material for: Genome-Wide Association Study for Traits Related to Plant and Grain Morphology, and Root Architecture in Temperate Rice Accessions
Source: PLoS One. 2016 May 26;11(5):e0155425. doi: 10.1371/journal.pone.0155425 (PMC4881974; doi:10.1371/journal.pone.0155425)
Supplement: S1 Table — List of accessions used in the study with their geographical origin, commercial class, taxonomical group and collected pheno- types (153 accessions phenotyped for plant and grain morphology; a subset of 93 accessions phenotyped also for root traits). (PDF) [file pone.0155425.s003.pdf]

Genome-wide association study for traits related to plant and grain morphology, and root architecture in temperate rice accessions

Filippo Biscarini[1,*] et al.

1 Department of Bioinformatics and Biostatistics, PTP Science Park, Lodi, Italy
∗ E-mail: filippo.biscarini@ptp.it

Table 1: List of accessions used in the study with their geographical origin, commercial class, taxonomical group and collected phenotypes (153 accessions phenotyped for plant and grain morphology; a subset of 93 accessions phenotyped also for root traits).

Name	Origin	Class	Group	Phenotypes 153	Phenotypes 93
9311			indica		
A201	Long B	USA	tropical_japonica	Plant/grain	Root
A301	Long B	USA	tropical_japonica	Plant/grain	
ADAIR	Long B	USA	tropical_japonica		
AGOSTANO	Long A	ITALIA	temperate_japonica		
AIACE	Long A	ITALIA	tropical_japonica	Plant/grain	
AKITAKOMACHI	Round	GIAPPONE	temperate_japonica	Plant/grain	Root
ALAN	Long B	USA	tropical_japonica		
ALEXANDROS	Long B	GRECIA	tropical_japonica		
ALLORIO	Long A	ITALIA	temperate_japonica	Plant/grain	Root
ALPE	Long A	ITALIA	temperate_japonica	Plant/grain	Root
ALPHA	Long A	ITALIA	temperate_japonica		
AMBRA		ITALIA	temperate_japonica		
AMERICANO_1600	Round	ITALIA	temperate_japonica	Plant/grain	
ANTONI	Long A	BULGARIA	temperate_japonica		
APOLLO	Long B	ITALIA	tropical_japonica	Plant/grain	Root
ARBORIO	Long A	ITALIA	temperate_japonica	Plant/grain	Root
ARC_10352			aromatic		
ARGO	Medium	ITALIA	temperate_japonica	Plant/grain	Root
ARIANA		ROMANIA	indica		
Arias			tropical_japonica		
ARIETE	Long A	ITALIA	temperate_japonica	Plant/grain	Root
ARLESIENNE		FRANCIA	temperate_japonica		
ARSENAL	Long B	ITALIA	tropical_japonica		
ASIA	Long B	ITALIA	aromatic	Plant/grain	
Asse_Y_Pung			tropical_japonica		
AUGUSTO	Long A	ITALIA	temperate_japonica	Plant/grain	Root
AUZGUSTA		UNGHERIA	temperate_japonica		
Azucena		USA	tropical_japonica		
BACCO	Long A	ITALIA	temperate_japonica		
BAHIA	Medium	SPAGNA	temperate_japonica		
BAIXET	Long A	SPAGNA	temperate_japonica		
BALILLA	Round	ITALIA	temperate_japonica	Plant/grain	Root
BALILLONE_R253			temperate_japonica		
BALOCCO_G	Medium	ITALIA	temperate_japonica		
BALZARETTI	Medium	ITALIA	temperate_japonica	Plant/grain	
BARAGGIA	Round	ITALIA	temperate_japonica	Plant/grain	Root
Basmati	Long B	INDIA	aromatic		
Basmati_1	Long B	INDIA	aus		
BASMATI_C621	Long B	INDIA	aromatic		
BEIRAO	Long A	PORTOGALLO	temperate_japonica	Plant/grain	Root
BELLE_PATNA	Long B	USA	tropical_japonica		
BERTONE	Long A	ITALIA	temperate_japonica	Plant/grain	Root
BETIS			temperate_japonica		
Bico_Branco			aromatic		

Binulawan-F			indica		
BJ_1			aus		
BLACK_EGYPT			temperate_japonica		
Black_Gora			aus		
BLUE_BONNET		USA	tropical_japonica		
BOMBA	Medium	SPAGNA	temperate_japonica		
BOMBILLA	Medium	SPAGNA	temperate_japonica		
BOMBON	Medium	SPAGNA	temperate_japonica		
BOND		USA	tropical_japonica		
BRAZOS	Long A	USA	tropical_japonica		
BRIO	Round	ITALIA	temperate_japonica		
BURMA	Long A	ITALIA	tropical_japonica		
CADET	Long B	ITALIA	tropical_japonica		
CALMOCHI_101	Medium	USA	temperate_japonica	Plant/grain	Root
CAMPINO	Medium	PORTOGALLO	temperate_japonica		
CAPATAZ	Long A	SPAGNA	temperate_japonica	Plant/grain	
CARINA	Round	BULGARIA	temperate_japonica		
CARMEN	Long A	ITALIA	temperate_japonica		
CARNAROLI	Long A	ITALIA	temperate_japonica	Plant/grain	Root
CARNISE	Long A	ITALIA	temperate_japonica		
CARNISE_PRECOCE	Long A	ITALIA	temperate_japonica		
CARRICO	Round	PORTOGALLO	temperate_japonica		
CASTELMOCHI	Round	ITALIA	temperate_japonica	Plant/grain	Root
CENTAURO	Round	ITALIA	temperate_japonica	Plant/grain	Root
CENTURY_PATNA		USA	tropical_japonica		
Chau			indica		
Chiem_Chanh			indica		
Chinese			temperate_japonica		
CHIPKA	Round	BULGARIA	temperate_japonica		
CIGALON	Medium	FRANCIA	temperate_japonica	Plant/grain	
CINIA_40		CILE	temperate_japonica	Plant/grain	Root
CLOT	Medium	SPAGNA	temperate_japonica		
CNA_4081	Long B	BRASILE	indica		
COCODRIE	Long B	USA	tropical_japonica	Plant/grain	
COLINA	Round	SPAGNA	temperate_japonica	Plant/grain	
CORBETTA	Medium	ITALIA	temperate_japonica		
CRIPTO		ITALIA	temperate_japonica	Plant/grain	Root
CRLB1	Long B	ITALIA	tropical_japonica		
CT58	Long A	COLOMBIA	temperate_japonica	Plant/grain	
Dee_Geo_Woo_Gen			indica		
DELFINO	Long A	ITALIA	temperate_japonica	Plant/grain	Root
DELLROSE	Long A	USA	tropical_japonica	Plant/grain	Root
DELTA		SPAGNA	temperate_japonica		
Dhala_Shaitta			aus		
DIMITRA	Long A	GRECIA	temperate_japonica		
DIXIEBELLE	Long A	USA	tropical_japonica	Plant/grain	
Dom_Sofid			aromatic		
DRAGO	Long A	ITALIA	temperate_japonica	Plant/grain	Root
DREW	Long B	USA	tropical_japonica	Plant/grain	Root
DUCATO	Round	ITALIA	temperate_japonica		
DV85			aus		
ELIO		ITALIA	temperate_japonica		
EOLO		ITALIA	tropical_japonica		
ERCOLE	Long A	ITALIA	temperate_japonica	Plant/grain	Root
ESCARLATE	Round	PORTOGALLO	temperate_japonica		
ESTRELA_IRRADIADO	Long A	PORTOGALLO	temperate_japonica		
EUROPA	Long A	ITALIA	temperate_japonica	Plant/grain	Root
Firooz			aromatic		
FLIPPER	Long B	ITALIA	temperate_japonica		
FORTUNA	Long A	ITALIA	tropical_japonica	Plant/grain	Root

FRAGRANCE	Long B	ITALIA	aromatic	Plant/grain	
FRANCES	Medium	SPAGNA	temperate_japonica		
FULGENTE	Medium	ITALIA	temperate_japonica	Plant/grain	
GANGE	Long B	ITALIA	tropical_japonica	Plant/grain	
GARDE_SADRI	Long A	TURCHIA	temperate_japonica		
Geumbyeo			temperate_japonica		
GIADA	Long B	ITALIA	tropical_japonica	Plant/grain	Root
GIGANTE_VERCELLI	Long A	ITALIA	temperate_japonica	Plant/grain	Root
GIOVANNI_MARCHETTI	Medium	ITALIA	temperate_japonica		
GIZA_177	Medium	EGITTO	temperate_japonica	Plant/grain	
GIZA_178	Round	EGITTO	indica	Plant/grain	
GLADIO	Long B	ITALIA	tropical_japonica	Plant/grain	Root
GUADIAMAR	Medium	SPAGNA	temperate_japonica		
Guan-Yin-Tsan			indica		
GZ6296	Long A	EGITTO	indica	Plant/grain	
GZ8367		EGITTO	temperate_japonica	Plant/grain	
HANDAO_11	Round	CINA	temperate_japonica	Plant/grain	Root
HANDAO_297	Round	CINA	temperate_japonica	Plant/grain	Root
HARRA	Round	AUSTRALIA	temperate_japonica		
HONDURAS	Long A	SPAGNA	tropical_japonica	Plant/grain	
IAC32_52	Long B	BRASILE	tropical_japonica		
IBO_380-33	Long A	PORTOGALLO	temperate_japonica	Plant/grain	
IBO_400	Long A	PORTOGALLO	temperate_japonica		
ILANG_ILANG		COREA DEL SUD	tropical_japonica		
IR64	Long B	FILIPPINE	indica	Plant/grain	
ITALMOCHI	Medium	ITALIA	temperate_japonica	Plant/grain	Root
ITALPATNA_48	Long A	ITALIA	temperate_japonica	Plant/grain	Root
JACINTO	Long A	USA	tropical_japonica		
Jaya			indica		
JEFFERSON	Long A	USA	tropical_japonica	Plant/grain	Root
Jhona_349			aus		
JUBILIENI	Round	BULGARIA	temperate_japonica		
KARNAK	Long A	ITALIA	temperate_japonica	Plant/grain	
Kasalath			aus		
KATY		USA	tropical_japonica		
Khao_Gaew			aus		
KING	Long B/Long A	ITALIA	tropical_japonica		
Kitrana_508			aromatic		
KORAL	Long A	ITALIA	temperate_japonica	Plant/grain	Root
Koshihikari			temperate_japonica		
KULON	Long A	RUSSIA	temperate_japonica	Plant/grain	
KYEEMA	Long B	AUSTRALIA	tropical_japonica		
L201	Long B	USA	tropical_japonica	Plant/grain	Root
L202	Long B	USA	tropical_japonica		
L204	Long B	USA	tropical_japonica		
L205	Long B	USA	tropical_japonica		
LACASSINE	Long B	USA	tropical_japonica		
LADY_WRIGHT	Medium	USA	tropical_japonica	Plant/grain	Root
LAGRUE	Long A	USA	tropical_japonica	Plant/grain	Root
LAMONE	Long B	ITALIA	tropical_japonica	Plant/grain	Root
Lemont		USA	tropical_japonica		
LENCINO	Round	ITALIA	temperate_japonica		
LIBERO		ITALIA	tropical_japonica		
LIDO	Medium	ITALIA	temperate_japonica	Plant/grain	Root
LOMELLINO	Medium	ITALIA	temperate_japonica	Plant/grain	
LOTO	Long A	ITALIA	temperate_japonica	Plant/grain	Root
LUCERO	Round	ITALIA	temperate_japonica	Plant/grain	Root
LUNA	Medium	USA	temperate_japonica		
LUSITO_IRRADIADO	Long A	PORTOGALLO	temperate_japonica		
LUXOR	Long A	ITALIA	temperate_japonica	Plant/grain	Root

M202	Medium	USA	temperate_japonica	Plant/grain	
M203	Long A	USA	temperate_japonica		
M204	Long A	USA	temperate_japonica	Plant/grain	Root
Mansaku			temperate_japonica		
MARATELLI	Medium	ITALIA	temperate_japonica	Plant/grain	Root
MARENY	Long A	SPAGNA	temperate_japonica		
MARISMA		SPAGNA	temperate_japonica		
MARTA		ITALIA	tropical_japonica		
MARTE	Round	ITALIA	temperate_japonica	Plant/grain	Root
MAYBELLE	Long B	USA	tropical_japonica		
MERCURIO	Long B	ITALIA	tropical_japonica		
MERLE	Long B	FRANCIA	indica		
MILEV_21	Round	BULGARIA	temperate_japonica		
Miriti			tropical_japonica		
Miriti-S2			tropical_japonica		
MONTICELLI	Medium	ITALIA	temperate_japonica	Plant/grain	Root
Moroberekan			tropical_japonica		
Mudgo			indica		
MUGA	Round	PORTOGALLO	temperate_japonica		
NEMBO	Long A	ITALIA	temperate_japonica	Plant/grain	Root
NILO	Long A	ITALIA	temperate_japonica		
Nipponbare		USA	temperate_japonica		
Norin_20			temperate_japonica		
NOVARA	Medium	ITALIA	temperate_japonica	Plant/grain	
OLYMPIADA	Long B	GRECIA	indica		
OPALE	Long A	ITALIA	temperate_japonica	Plant/grain	
ORIGINARIO	Round	ITALIA	temperate_japonica		
ORIONE	Long A	ITALIA	temperate_japonica	Plant/grain	Root
OSTIGLIA	Round	ITALIA	temperate_japonica	Plant/grain	
OTA	Long A	PORTOGALLO	temperate_japonica	Plant/grain	Root
P6	Medium	ITALIA	temperate_japonica		
PADANO	Long A	ITALIA	temperate_japonica	Plant/grain	Root
PEGONIL	Medium	SPAGNA	temperate_japonica		
PELDE		AUSTRALIA	temperate_japonica	Plant/grain	
PERLA	Round	ITALIA	temperate_japonica	Plant/grain	Root
Phudugey			aus		
PIEMONTE	Long A	ITALIA	temperate_japonica	Plant/grain	Root
PLOVDIV_22	Long A	BULGARIA	temperate_japonica	Plant/grain	Root
PLOVDIV_24	Round	BULGARIA	temperate_japonica		
PLUS	Long B	ITALIA	tropical_japonica		
POLIZESTI_28		ROMANIA	temperate_japonica		
POSEIDONE	Medium	ITALIA	temperate_japonica		
Pratao			tropical_japonica		
PRECOCE_6			temperate_japonica		
PRECOCE_ROSSI			temperate_japonica		
PRECOZ_2FA		ARGENTINA	temperate_japonica		
PROMETEO	Medium	ITALIA	temperate_japonica	Plant/grain	Root
PUNTAL	Long B	SPAGNA	tropical_japonica		
R_271			temperate_japonica		
RADON		ITALIA	temperate_japonica		
RANGHINO	Round	ITALIA	temperate_japonica	Plant/grain	Root
Rathuwee			indica		
RAZZA_77	Medium	ITALIA	temperate_japonica	Plant/grain	
RB_GAMMA			temperate_japonica		
REXMONT	Long B	USA	tropical_japonica		
RIBE	Long A	ITALIA	temperate_japonica	Plant/grain	
RIBE_JAUNE			temperate_japonica		
RINALDO_BERSANI	Long A	ITALIA	temperate_japonica	Plant/grain	Root
RINGO	Long A	ITALIA	temperate_japonica	Plant/grain	
ROBBIO_SEL1	Long A	ITALIA	temperate_japonica	Plant/grain	Root

RODEO	Long A	ITALIA	temperate_japonica	Plant/grain	Root
RODINA	Round	BULGARIA	temperate_japonica	Plant/grain	
ROMA	Long A	ITALIA	temperate_japonica	Plant/grain	
ROTUNDUS	Long A	UNGHERIA	temperate_japonica		
ROXANI	Long A	GRECIA	temperate_japonica	Plant/grain	
RPC_12	Round	CINA	temperate_japonica		
RUSSO		ITALIA	temperate_japonica	Plant/grain	
S101	Medium	USA	temperate_japonica		
S102	Medium	USA	temperate_japonica		
S102_2	Medium	USA	temperate_japonica		
SAEDINENIE	Long A	BULGARIA	temperate_japonica		
SAFARI	Long A	PORTOGALLO	temperate_japonica	Plant/grain	
SAKHA_102	Medium	EGITTO	temperate_japonica	Plant/grain	Root
SAKHA_103	Round	EGITTO	temperate_japonica		
SALOIO	Long B	PORTOGALLO	temperate_japonica		
SANDORA	Long A	UNGHERIA	temperate_japonica		
SANT_ANDREA	Long A	ITALIA	temperate_japonica	Plant/grain	Root
SATURNO	Long B	ITALIA	tropical_japonica	Plant/grain	
SCUDO	Long B	ITALIA	tropical_japonica	Plant/grain	Root
SELENIO	Round	ITALIA	temperate_japonica	Plant/grain	Root
SELN_244A620	Medium	AUSTRALIA	temperate_japonica		
SENIA	Medium	SPAGNA	temperate_japonica	Plant/grain	
SEQUIAL	Medium	SPAGNA	temperate_japonica		
SESIAMOCHI	Long A/Round	ITALIA	temperate_japonica	Plant/grain	
SETANTUNO	Round	PORTOGALLO	temperate_japonica	Plant/grain	
SHANGHAI	Long A	CINA	aus		
Shinrike			temperate_japonica		
Shoemed			temperate_japonica		
SHSS_381	Long A	SPAGNA	temperate_japonica		
SHSS_53	Long A	SPAGNA	temperate_japonica		
SIRIO_CL	Long A	ITALIA	tropical_japonica		
SIS_R215	Long A	ITALIA	tropical_japonica	Plant/grain	Root
SLAVA	Medium	BULGARIA	temperate_japonica		
SMERALDO	Long A	ITALIA	temperate_japonica	Plant/grain	Root
SP55			temperate_japonica		
SPRINT	Long B	ITALIA	tropical_japonica		
SR_113	Long A	SPAGNA	temperate_japonica		
Suweon_362		KOREA	temperate_japonica		
T_1			aus		
T757		INDIA	temperate_japonica		
Taducan			indica		
TAICHUNG_65		THAILANDIA	temperate_japonica		
TEA	Medium	ITALIA	temperate_japonica		
TEJO	Long A	ITALIA	temperate_japonica	Plant/grain	Root
TEQING	Long B	CINA	indica	Plant/grain	
TEXMONT	Long A	USA	tropical_japonica		
THAIBONNET	Long B	ITALIA	tropical_japonica	Plant/grain	
THAIPERLA			temperate_japonica		
TIMICH_108		ROMANIA	temperate_japonica		
TITANIO		ITALIA	temperate_japonica	Plant/grain	Root
TITANO (ATLANTE)	Long B	ITALIA	tropical_japonica		
TOPAZIO	Medium	ITALIA	temperate_japonica		
TORIO	Long A	PORTOGALLO	temperate_japonica		
Trembese			tropical_japonica		
ULISSE	Long A	ITALIA	temperate_japonica	Plant/grain	Root
ULLAL	Round	SPAGNA	temperate_japonica		
UPLA_77	Long B	ARGENTINA	tropical_japonica	Plant/grain	Root
UPLA_79	Long B	ARGENTINA	tropical_japonica		
UPLA_91	Long B	ARGENTINA	tropical_japonica	Plant/grain	
URANO	Long B	ITALIA	tropical_japonica		

VALTEJO	Round	PORTOGALLO	temperate_japonica	Plant/grain	
VARIETA_16			temperate_japonica		
VENERE	Long B	ITALIA	temperate_japonica	Plant/grain	Root
VIALE	Long A	ITALIA	temperate_japonica		
VIALONE_190	Medium	ITALIA	temperate_japonica	Plant/grain	Root
VIALONE_NANO	Medium	ITALIA	temperate_japonica	Plant/grain	Root
VICTORIA	Round	ARGENTINA	temperate_japonica	Plant/grain	
VIRGO	Medium	ITALIA	temperate_japonica		
VOLANO	Long A	ITALIA	temperate_japonica	Plant/grain	Root
YRL_196		AUSTRALIA	temperate_japonica		
YRM_6_2	Medium	AUSTRALIA	temperate_japonica		
ZENITH	Medium	USA	tropical_japonica		
ZHENSHANG_97		CINA	indica	Plant/grain	
RUBINO	Round	ITALIA	temperate_japonica		
RONCOLO	Medium	ITALIA	temperate_japonica		
OLCENENGO	Long A	ITALIA	temperate_japonica		
SOURE	Long A	PORTOGALLO	temperate_japonica		
NIBBIO	Medium	ITALIA	temperate_japonica		
REDI	Long A	ITALIA	temperate_japonica	Plant/grain	Root
VENERIA	Long A	ITALIA	temperate_japonica		
BALDO	Long A	ITALIA	temperate_japonica	Plant/grain	Root
STRELLA	Long A	ITALIA	temperate_japonica		
CERVO		ITALIA	temperate_japonica		
ONDA		ITALIA	temperate_japonica		
M6	Long A	ITALIA	temperate_japonica		
ADELAIDE_CHIAPPELLI	Long A	ITALIA	temperate_japonica	Plant/grain	
SILLA	Long A	ITALIA	temperate_japonica	Plant/grain	Root
MOLO	Long A	ITALIA	temperate_japonica	Plant/grain	
CALENDAL	Long A	FRANCIA	temperate_japonica		
TOSCA	Long A	ITALIA	temperate_japonica		
PIERINA_MARCHETTI	Long A	ITALIA	temperate_japonica		
RIZZOTTO_51_1	Long A	ITALIA	temperate_japonica		
SESIA	Long A	ITALIA	temperate_japonica	Plant/grain	
ALICE	Long A	ITALIA	temperate_japonica	Plant/grain	Root
MANTOVA	Long A	ITALIA	temperate_japonica	Plant/grain	
FAMILIA_181	Long A	PORTOGALLO	temperate_japonica		
FAISCA			temperate_japonica		
BONNI	Long A	ITALIA	temperate_japonica		
SUPER		PORTOGALLO	temperate_japonica	Plant/grain	Root
EUROSE	Long A	ITALIA	temperate_japonica		
GRITNA	Long A	ITALIA	temperate_japonica	Plant/grain	
BORRACHO			temperate_japonica		
SAVIO	Long A	ITALIA	temperate_japonica	Plant/grain	
HAREM	Long A	PORTOGALLO	temperate_japonica		
ARTICO			indica		
SENATORE_NOVELLI	Long A	ITALIA	temperate_japonica		
RUBIDIO			temperate_japonica		
RUBI		PORTOGALLO	temperate_japonica	Plant/grain	
RIVA		ITALIA	temperate_japonica		
LORD	Long A	ITALIA	temperate_japonica	Plant/grain	Root
ZENA	Long B	ITALIA	tropical_japonica	Plant/grain	Root
SANDOCA	Long B	PORTOGALLO	temperate_japonica		
GALILEO	Long A	ITALIA	temperate_japonica	Plant/grain	
CT36	Long B	COLOMBIA	temperate_japonica		
ARTIGLIO	Long B	ITALIA	indica		
GHIBLI		ITALIA	temperate_japonica		
CRESO	Long A	ITALIA	temperate_japonica	Plant/grain	
UPLA_75	Long B	ARGENTINA	tropical_japonica	Plant/grain	Root
SCIROCCO	Medium	ITALIA	temperate_japonica		
UPLA_32	Long B	ARGENTINA	tropical_japonica	Plant/grain	Root

UPLA_80	Long B	ARGENTINA	tropical_japonica		
BASMATI_DETRADUNI	Long B	INDIA	indica		
BIANCA	Long A	ITALIA	temperate_japonica	Plant/grain	Root
VELA	Long A	ITALIA	temperate_japonica		
MELAS	Long B	GRECIA	temperate_japonica	Plant/grain	Root
UPLA_63	Long B	ARGENTINA	tropical_japonica	Plant/grain	
TARRISO	Long B	ITALIA	tropical_japonica		
UPLA_104	Long B	ARGENTINA	tropical_japonica		
PANDA		ITALIA	tropical_japonica	Plant/grain	Root
SAGRES	Long A	PORTOGALLO	temperate_japonica		
UPLA_66	Long B	ARGENTINA	tropical_japonica		
MIARA	Long B	ITALIA	temperate_japonica		
UPLA_68	Long B	ARGENTINA	tropical_japonica	Plant/grain	
CARIOCA	Long B	ITALIA	tropical_japonica		
SAMBA	Long A	ITALIA	tropical_japonica		
GRAAL	Long B	FRANCIA	tropical_japonica		
CHACARERO			temperate_japonica		
ALINANO		FRANCIA	temperate_japonica		
ELLEBI	Long B	ITALIA	tropical_japonica		
NANO	Round	ITALIA	temperate_japonica		
CT23		COLOMBIA	temperate_japonica		
MEJANES	Long B	FRANCIA	temperate_japonica		
GIANO	Long B	ITALIA	tropical_japonica	Plant/grain	Root
ITALPATNAxMILYANG	Long A	PORTOGALLO	temperate_japonica		
BAJANGxALLORIO			temperate_japonica		
SALVO	Long B	ITALIA	tropical_japonica	Plant/grain	Root
DARDO	Long A	ITALIA	tropical_japonica		
EUROSIS	Long A	ITALIA	temperate_japonica	Plant/grain	Root
SANTERNO	Long B	ITALIA	temperate_japonica	Plant/grain	Root
ALBATROS		ITALIA	temperate_japonica		
GREPPI	Round	ITALIA	tropical_japonica	Plant/grain	Root
ESTRELA	Long A	PORTOGALLO	temperate_japonica		
UPLA_64	Long B	ARGENTINA	tropical_japonica		
MAIORAL	Long A	PORTOGALLO	temperate_japonica		
PREVER	Long B	ITALIA	tropical_japonica		
ERMES	Long B	ITALIA	tropical_japonica	Plant/grain	
PECOS	Medium	USA	tropical_japonica	Plant/grain	Root
ANSEATICO	Long A	ITALIA	temperate_japonica		
ARTEMIDE	Long B	ITALIA	tropical_japonica	Plant/grain	Root
BENGAL	Long A	USA	temperate_japonica	Plant/grain	
MIDA			temperate_japonica		
GRALDO	Long B	ITALIA	tropical_japonica	Plant/grain	Root
RONALDO	Long A	ITALIA	tropical_japonica		
ROMBO	Long A	ITALIA	temperate_japonica		
OSCARxSUWEON	Long A	PORTOGALLO	temperate_japonica		
FIDJI	Long B	FILIPPINE	tropical_japonica		
