# Supplementary material for: Genome-Wide Association Study for Traits Related to Plant and Grain Morphology, and Root Architecture in Temperate Rice Accessions
Source: PLoS One. 2016 May 26;11(5):e0155425. doi: 10.1371/journal.pone.0155425 (PMC4881974; doi:10.1371/journal.pone.0155425)
Supplement: S2 Table — Command lines and parameters used to generate the imputed non-filtered SNP dataset with Tassel GBS pipeline 3.0. Command lines with the “Plugin” suffix are part of the TASSEL pipeline; filenames and directories are not listed. In order to run the “FastImputationBitFixedWindowPlugin”, a tab-separated file with an inbreeding coefficient of 0.99 for each taxon was provided. When not reported, default parameters were used. (PDF) [file pone.0155425.s004.pdf]

Genome-wide association study for traits related to plant and grain morphology, and root architecture in temperate rice accessions

Filippo Biscarini[1,*] et al.
1 Department of Bioinformatics and Biostatistics, PTP Science Park, Lodi, Italy
*** E-mail: filippo.biscarini@ptp.it**

Table 1. Command lines and parameters used to generate the imputed non-filtered SNP dataset with Tassel GBS pipeline 3.0. Command lines with the Plugin suffix are part of the TASSEL pipeline; filenames and directories are not listed. In order to run the FastImputationBitFixedWindowPlugin, a tab-separated file with an inbreeding coefficient of 0.99 for each taxon was provided. When not reported, default parameters were used.

Command line	Parameters
FastqToTagCountPlugin	-Xmx16G -i fastq -e ApeKI -s 400000000
MergeMultipleTagCountPlugin	-c 5 -t
bwa index	-a is
bwa aln	-t 8
bwa samse	
SAMConverterPlugin	
FastqToTBTPlugin	-Xmx16G -i fastq -e ApeKI -y -s 400000000
MergeTagsByTaxaFilesPlugin	-Xmx16G -s 400000000
TagsToSNPByAlignmentPlugin	-mnF 0.99 -mnMAF 0.01 -mnMAC 10 -s 1 -e 12 -y
MergeDuplicateSNPsPlugin	-s 1 -e 12
FastImputationBitFixedWindowPlugin	
