# Supplementary material for: Genome-Wide Association Study for Traits Related to Plant and Grain Morphology, and Root Architecture in Temperate Rice Accessions
Source: PLoS One. 2016 May 26;11(5):e0155425. doi: 10.1371/journal.pone.0155425 (PMC4881974; doi:10.1371/journal.pone.0155425)
Supplement: S3 Table — List of accessions used in the study with the taxonomical group they have been assigned to and the probability of assignment to each of the 5 major groups based on their SNP genotypes. (PDF) [file pone.0155425.s005.pdf]

Genome-wide association study for traits related to plant and grain morphology, and root architecture in temperate rice accessions

Filippo Biscarini[1,*] et al.

1 Department of Bioinformatics and Biostatistics, PTP Science Park, Lodi, Italy
*** E-mail: filippo.biscarini@ptp.it**

Table 1: List of accessions used in the study with the taxonomical group they have been assigned to and the probability of assignment to each of the 5 major groups based on their SNP genotypes.

Accession	Group_Admixture	Score_Admixture	indica	trop_jap	temp_jap	aromatic	aus
9311	indica	0.99996	0.99996	0.00001	0.00001	0.00001	0.00001
A201	tropical_japonica	0.99996	0.00001	0.99996	0.00001	0.00001	0.00001
A301	tropical_japonica	0.99996	0.00001	0.99996	0.00001	0.00001	0.00001
ADAIR	tropical_japonica	0.99996	0.00001	0.99996	0.00001	0.00001	0.00001
AGOSTANO	temperate_japonica	0.99996	0.00001	0.00001	0.99996	0.00001	0.00001
AIACE	tropical_japonica	0.99996	0.00001	0.99996	0.00001	0.00001	0.00001
AKITAKOMACHI	temperate_japonica	0.99996	0.00001	0.00001	0.99996	0.00001	0.00001
ALAN	tropical_japonica	0.99996	0.00001	0.99996	0.00001	0.00001	0.00001
ALEXANDROS	tropical_japonica	0.99996	0.00001	0.99996	0.00001	0.00001	0.00001
ALLORIO	temperate_japonica	0.99996	0.00001	0.00001	0.99996	0.00001	0.00001
ALPE	temperate_japonica	0.99996	0.00001	0.00001	0.99996	0.00001	0.00001
ALPHA	temperate_japonica	0.99996	0.00001	0.00001	0.99996	0.00001	0.00001
AMBRA	temperate_japonica	0.99996	0.00001	0.00001	0.99996	0.00001	0.00001
AMERICANO_1600	temperate_japonica	0.99996	0.00001	0.00001	0.99996	0.00001	0.00001
ANTONI	temperate_japonica	0.99996	0.00001	0.00001	0.99996	0.00001	0.00001
APOLLO	tropical_japonica	0.99996	0.00001	0.99996	0.00001	0.00001	0.00001
ARBORIO	temperate_japonica	0.99996	0.00001	0.00001	0.99996	0.00001	0.00001
ARC_10352	aromatic	0.99996	0.00001	0.00001	0.00001	0.99996	0.00001
ARGO	temperate_japonica	0.99996	0.00001	0.00001	0.99996	0.00001	0.00001
ARIANA	indica	0.99996	0.99996	0.00001	0.00001	0.00001	0.00001
Arias	tropical_japonica	0.99996	0.00001	0.99996	0.00001	0.00001	0.00001
ARIETE	temperate_japonica	0.99996	0.00001	0.00001	0.99996	0.00001	0.00001
ARLESIENNE	temperate_japonica	0.99996	0.00001	0.00001	0.99996	0.00001	0.00001
ARSENAL	tropical_japonica	0.99996	0.00001	0.99996	0.00001	0.00001	0.00001
ASIA	aromatic	0.99996	0.00001	0.00001	0.00001	0.99996	0.00001
Asse_Y_Pung	tropical_japonica	0.99996	0.00001	0.99996	0.00001	0.00001	0.00001
AUGUSTO	temperate_japonica	0.99996	0.00001	0.00001	0.99996	0.00001	0.00001
AUZGUSTA	temperate_japonica	0.99996	0.00001	0.00001	0.99996	0.00001	0.00001
Azucena	tropical_japonica	0.99996	0.00001	0.99996	0.00001	0.00001	0.00001
BACCO	temperate_japonica	0.99996	0.00001	0.00001	0.99996	0.00001	0.00001
BAHIA	temperate_japonica	0.99996	0.00001	0.00001	0.99996	0.00001	0.00001
BAIXET	temperate_japonica	0.99996	0.00001	0.00001	0.99996	0.00001	0.00001
BALILLA	temperate_japonica	0.99996	0.00001	0.00001	0.99996	0.00001	0.00001
BALILLONE_R253	temperate_japonica	0.99996	0.00001	0.00001	0.99996	0.00001	0.00001
BALOCCO_G	temperate_japonica	0.99996	0.00001	0.00001	0.99996	0.00001	0.00001
BALZARETTI	temperate_japonica	0.99996	0.00001	0.00001	0.99996	0.00001	0.00001
BARAGGIA	temperate_japonica	0.99996	0.00001	0.00001	0.99996	0.00001	0.00001
Basmati	aromatic	0.99996	0.00001	0.00001	0.00001	0.99996	0.00001
Basmati_1	aus	0.99996	0.00001	0.00001	0.00001	0.00001	0.99996
BASMATI_C621	aromatic	0.99996	0.00001	0.00001	0.00001	0.99996	0.00001
BEIRAO	temperate_japonica	0.99996	0.00001	0.00001	0.99996	0.00001	0.00001
BELLE_PATNA	tropical_japonica	0.99996	0.00001	0.99996	0.00001	0.00001	0.00001
BERTONE	temperate_japonica	0.99996	0.00001	0.00001	0.99996	0.00001	0.00001
BETIS	temperate_japonica	0.99996	0.00001	0.00001	0.99996	0.00001	0.00001
Bico_Branco	aromatic	0.99996	0.00001	0.00001	0.00001	0.99996	0.00001
Binulawan-F	indica	0.99996	0.99996	0.00001	0.00001	0.00001	0.00001

BJ_1	aus	0.99996	0.00001	0.00001	0.00001	0.00001	0.99996
BLACK_EGYPT	temperate_japonica	0.99996	0.00001	0.00001	0.99996	0.00001	0.00001
Black_Gora	aus	0.99996	0.00001	0.00001	0.00001	0.00001	0.99996
BLUE_BONNET	tropical_japonica	0.99996	0.00001	0.99996	0.00001	0.00001	0.00001
BOMBA	temperate_japonica	0.99996	0.00001	0.00001	0.99996	0.00001	0.00001
BOMBILLA	temperate_japonica	0.99996	0.00001	0.00001	0.99996	0.00001	0.00001
BOMBON	temperate_japonica	0.99996	0.00001	0.00001	0.99996	0.00001	0.00001
BOND	tropical_japonica	0.99996	0.00001	0.99996	0.00001	0.00001	0.00001
BRAZOS	tropical_japonica	0.99996	0.00001	0.99996	0.00001	0.00001	0.00001
BRIO	temperate_japonica	0.99996	0.00001	0.00001	0.99996	0.00001	0.00001
BURMA	tropical_japonica	0.99996	0.00001	0.99996	0.00001	0.00001	0.00001
CADET	tropical_japonica	0.99996	0.00001	0.99996	0.00001	0.00001	0.00001
CALMOCHI_101	temperate_japonica	0.99996	0.00001	0.00001	0.99996	0.00001	0.00001
CAMPINO	temperate_japonica	0.99996	0.00001	0.00001	0.99996	0.00001	0.00001
CAPATAZ	temperate_japonica	0.99996	0.00001	0.00001	0.99996	0.00001	0.00001
CARINA	temperate_japonica	0.99996	0.00001	0.00001	0.99996	0.00001	0.00001
CARMEN	temperate_japonica	0.99996	0.00001	0.00001	0.99996	0.00001	0.00001
CARNAROLI	temperate_japonica	0.99996	0.00001	0.00001	0.99996	0.00001	0.00001
CARNISE	temperate_japonica	0.99996	0.00001	0.00001	0.99996	0.00001	0.00001
CARNISE_PRECOCE	temperate_japonica	0.99996	0.00001	0.00001	0.99996	0.00001	0.00001
CARRICO	temperate_japonica	0.99996	0.00001	0.00001	0.99996	0.00001	0.00001
CASTELMOCHI	temperate_japonica	0.99996	0.00001	0.00001	0.99996	0.00001	0.00001
CENTAURO	temperate_japonica	0.99996	0.00001	0.00001	0.99996	0.00001	0.00001
CENTURY_PATNA	tropical_japonica	0.99996	0.00001	0.99996	0.00001	0.00001	0.00001
Chau	indica	0.99996	0.99996	0.00001	0.00001	0.00001	0.00001
Chiem_Chanh	indica	0.99996	0.99996	0.00001	0.00001	0.00001	0.00001
Chinese	temperate_japonica	0.99996	0.00001	0.00001	0.99996	0.00001	0.00001
CHIPKA	temperate_japonica	0.99996	0.00001	0.00001	0.99996	0.00001	0.00001
CIGALON	temperate_japonica	0.99996	0.00001	0.00001	0.99996	0.00001	0.00001
CINIA_40	temperate_japonica	0.99996	0.00001	0.00001	0.99996	0.00001	0.00001
CLOT	temperate_japonica	0.99996	0.00001	0.00001	0.99996	0.00001	0.00001
CNA_4081	indica	0.99996	0.99996	0.00001	0.00001	0.00001	0.00001
COCODRIE	tropical_japonica	0.99996	0.00001	0.99996	0.00001	0.00001	0.00001
COLINA	temperate_japonica	0.99996	0.00001	0.00001	0.99996	0.00001	0.00001
CORBETTA	temperate_japonica	0.99996	0.00001	0.00001	0.99996	0.00001	0.00001
CRIPTO	temperate_japonica	0.99996	0.00001	0.00001	0.99996	0.00001	0.00001
CRLB1	tropical_japonica	0.99996	0.00001	0.99996	0.00001	0.00001	0.00001
CT58	temperate_japonica	0.99996	0.00001	0.00001	0.99996	0.00001	0.00001
Dee_Geo_Woo_Gen	indica	0.99996	0.99996	0.00001	0.00001	0.00001	0.00001
DELFINO	temperate_japonica	0.99996	0.00001	0.00001	0.99996	0.00001	0.00001
DELLROSE	tropical_japonica	0.99996	0.00001	0.99996	0.00001	0.00001	0.00001
DELTA	temperate_japonica	0.99996	0.00001	0.00001	0.99996	0.00001	0.00001
Dhala_Shaitta	aus	0.99996	0.00001	0.00001	0.00001	0.00001	0.99996
DIMITRA	temperate_japonica	0.99996	0.00001	0.00001	0.99996	0.00001	0.00001
DIXIEBELLE	tropical_japonica	0.99996	0.00001	0.99996	0.00001	0.00001	0.00001
Dom_Sofid	aromatic	0.99996	0.00001	0.00001	0.00001	0.99996	0.00001
DRAGO	temperate_japonica	0.99996	0.00001	0.00001	0.99996	0.00001	0.00001
DREW	tropical_japonica	0.99996	0.00001	0.99996	0.00001	0.00001	0.00001
DUCATO	temperate_japonica	0.99996	0.00001	0.00001	0.99996	0.00001	0.00001
DV85	aus	0.99996	0.00001	0.00001	0.00001	0.00001	0.99996
ELIO	temperate_japonica	0.99996	0.00001	0.00001	0.99996	0.00001	0.00001
EOLO	tropical_japonica	0.99996	0.00001	0.99996	0.00001	0.00001	0.00001
ERCOLE	temperate_japonica	0.99996	0.00001	0.00001	0.99996	0.00001	0.00001
ESCARLATE	temperate_japonica	0.99996	0.00001	0.00001	0.99996	0.00001	0.00001
ESTRELA_IRRADIADO	temperate_japonica	0.99996	0.00001	0.00001	0.99996	0.00001	0.00001
EUROPA	temperate_japonica	0.99996	0.00001	0.00001	0.99996	0.00001	0.00001
Firooz	aromatic	0.99996	0.00001	0.00001	0.00001	0.99996	0.00001
FLIPPER	temperate_japonica	0.99996	0.00001	0.00001	0.99996	0.00001	0.00001
FORTUNA	tropical_japonica	0.99996	0.00001	0.99996	0.00001	0.00001	0.00001
FRAGRANCE	aromatic	0.99996	0.00001	0.00001	0.00001	0.99996	0.00001

FRANCES	temperate_japonica	0.99996	0.00001	0.00001	0.99996	0.00001	0.00001
FULGENTE	temperate_japonica	0.99996	0.00001	0.00001	0.99996	0.00001	0.00001
GANGE	tropical_japonica	0.99996	0.00001	0.99996	0.00001	0.00001	0.00001
GARDE_SADRI	temperate_japonica	0.99996	0.00001	0.00001	0.99996	0.00001	0.00001
Geumbyeo	temperate_japonica	0.99996	0.00001	0.00001	0.99996	0.00001	0.00001
GIADA	tropical_japonica	0.99996	0.00001	0.99996	0.00001	0.00001	0.00001
GIGANTE_VERCELLI	temperate_japonica	0.99996	0.00001	0.00001	0.99996	0.00001	0.00001
GIOVANNI_MARCHETTI	temperate_japonica	0.99996	0.00001	0.00001	0.99996	0.00001	0.00001
GIZA_177	temperate_japonica	0.99996	0.00001	0.00001	0.99996	0.00001	0.00001
GIZA_178	indica	0.99996	0.99996	0.00001	0.00001	0.00001	0.00001
GLADIO	tropical_japonica	0.99996	0.00001	0.99996	0.00001	0.00001	0.00001
GUADIAMAR	temperate_japonica	0.99996	0.00001	0.00001	0.99996	0.00001	0.00001
Guan-Yin-Tsan	indica	0.99996	0.99996	0.00001	0.00001	0.00001	0.00001
GZ6296	indica	0.99996	0.99996	0.00001	0.00001	0.00001	0.00001
GZ8367	temperate_japonica	0.99996	0.00001	0.00001	0.99996	0.00001	0.00001
HANDAO_11	temperate_japonica	0.99996	0.00001	0.00001	0.99996	0.00001	0.00001
HANDAO_297	temperate_japonica	0.99996	0.00001	0.00001	0.99996	0.00001	0.00001
HARRA	temperate_japonica	0.99996	0.00001	0.00001	0.99996	0.00001	0.00001
HONDURAS	tropical_japonica	0.99996	0.00001	0.99996	0.00001	0.00001	0.00001
IAC32_52	tropical_japonica	0.99996	0.00001	0.99996	0.00001	0.00001	0.00001
IBO_380-33	temperate_japonica	0.99996	0.00001	0.00001	0.99996	0.00001	0.00001
IBO_400	temperate_japonica	0.99996	0.00001	0.00001	0.99996	0.00001	0.00001
ILANG_ILANG	tropical_japonica	0.99996	0.00001	0.99996	0.00001	0.00001	0.00001
IR64	indica	0.99996	0.99996	0.00001	0.00001	0.00001	0.00001
ITALMOCHI	temperate_japonica	0.99996	0.00001	0.00001	0.99996	0.00001	0.00001
ITALPATNA_48	temperate_japonica	0.99996	0.00001	0.00001	0.99996	0.00001	0.00001
JACINTO	tropical_japonica	0.99996	0.00001	0.99996	0.00001	0.00001	0.00001
Jaya	indica	0.99996	0.99996	0.00001	0.00001	0.00001	0.00001
JEFFERSON	tropical_japonica	0.99996	0.00001	0.99996	0.00001	0.00001	0.00001
Jhona_349	aus	0.99996	0.00001	0.00001	0.00001	0.00001	0.99996
JUBILIENI	temperate_japonica	0.99996	0.00001	0.00001	0.99996	0.00001	0.00001
KARNAK	temperate_japonica	0.99996	0.00001	0.00001	0.99996	0.00001	0.00001
Kasalath	aus	0.99996	0.00001	0.00001	0.00001	0.00001	0.99996
KATY	tropical_japonica	0.99996	0.00001	0.99996	0.00001	0.00001	0.00001
Khao_Gaew	aus	0.99996	0.00001	0.00001	0.00001	0.00001	0.99996
KING	tropical_japonica	0.99996	0.00001	0.99996	0.00001	0.00001	0.00001
Kitrana_508	aromatic	0.99996	0.00001	0.00001	0.00001	0.99996	0.00001
KORAL	temperate_japonica	0.99996	0.00001	0.00001	0.99996	0.00001	0.00001
Koshihikari	temperate_japonica	0.99996	0.00001	0.00001	0.99996	0.00001	0.00001
KULON	temperate_japonica	0.99996	0.00001	0.00001	0.99996	0.00001	0.00001
KYEEMA	tropical_japonica	0.99996	0.00001	0.99996	0.00001	0.00001	0.00001
L201	tropical_japonica	0.99996	0.00001	0.99996	0.00001	0.00001	0.00001
L202	tropical_japonica	0.99996	0.00001	0.99996	0.00001	0.00001	0.00001
L204	tropical_japonica	0.99996	0.00001	0.99996	0.00001	0.00001	0.00001
L205	tropical_japonica	0.99996	0.00001	0.99996	0.00001	0.00001	0.00001
LACASSINE	tropical_japonica	0.99996	0.00001	0.99996	0.00001	0.00001	0.00001
LADY_WRIGHT	tropical_japonica	0.99996	0.00001	0.99996	0.00001	0.00001	0.00001
LAGRUE	tropical_japonica	0.99996	0.00001	0.99996	0.00001	0.00001	0.00001
LAMONE	tropical_japonica	0.99996	0.00001	0.99996	0.00001	0.00001	0.00001
Lemont	tropical_japonica	0.99996	0.00001	0.99996	0.00001	0.00001	0.00001
LENCINO	temperate_japonica	0.99996	0.00001	0.00001	0.99996	0.00001	0.00001
LIBERO	tropical_japonica	0.99996	0.00001	0.99996	0.00001	0.00001	0.00001
LIDO	temperate_japonica	0.99996	0.00001	0.00001	0.99996	0.00001	0.00001
LOMELLINO	temperate_japonica	0.99996	0.00001	0.00001	0.99996	0.00001	0.00001
LOTO	temperate_japonica	0.99996	0.00001	0.00001	0.99996	0.00001	0.00001
LUCERO	temperate_japonica	0.99996	0.00001	0.00001	0.99996	0.00001	0.00001
LUNA	temperate_japonica	0.99996	0.00001	0.00001	0.99996	0.00001	0.00001
LUSITO_IRRADIADO	temperate_japonica	0.99996	0.00001	0.00001	0.99996	0.00001	0.00001
LUXOR	temperate_japonica	0.99996	0.00001	0.00001	0.99996	0.00001	0.00001
M202	temperate_japonica	0.99996	0.00001	0.00001	0.99996	0.00001	0.00001

M203	temperate_japonica	0.99996	0.00001	0.00001	0.99996	0.00001	0.00001
M204	temperate_japonica	0.99996	0.00001	0.00001	0.99996	0.00001	0.00001
Mansaku	temperate_japonica	0.99996	0.00001	0.00001	0.99996	0.00001	0.00001
MARATELLI	temperate_japonica	0.99996	0.00001	0.00001	0.99996	0.00001	0.00001
MARENY	temperate_japonica	0.99996	0.00001	0.00001	0.99996	0.00001	0.00001
MARISMA	temperate_japonica	0.99996	0.00001	0.00001	0.99996	0.00001	0.00001
MARTA	tropical_japonica	0.99996	0.00001	0.99996	0.00001	0.00001	0.00001
MARTE	temperate_japonica	0.99996	0.00001	0.00001	0.99996	0.00001	0.00001
MAYBELLE	tropical_japonica	0.99996	0.00001	0.99996	0.00001	0.00001	0.00001
MERCURIO	tropical_japonica	0.99996	0.00001	0.99996	0.00001	0.00001	0.00001
MERLE	indica	0.99996	0.99996	0.00001	0.00001	0.00001	0.00001
MILEV_21	temperate_japonica	0.99996	0.00001	0.00001	0.99996	0.00001	0.00001
Miriti	tropical_japonica	0.99996	0.00001	0.99996	0.00001	0.00001	0.00001
Miriti-S2	tropical_japonica	0.99996	0.00001	0.99996	0.00001	0.00001	0.00001
MONTICELLI	temperate_japonica	0.99996	0.00001	0.00001	0.99996	0.00001	0.00001
Moroberekan	tropical_japonica	0.99996	0.00001	0.99996	0.00001	0.00001	0.00001
Mudgo	indica	0.99996	0.99996	0.00001	0.00001	0.00001	0.00001
MUGA	temperate_japonica	0.99996	0.00001	0.00001	0.99996	0.00001	0.00001
NEMBO	temperate_japonica	0.99996	0.00001	0.00001	0.99996	0.00001	0.00001
NILO	temperate_japonica	0.99996	0.00001	0.00001	0.99996	0.00001	0.00001
Nipponbare	temperate_japonica	0.99996	0.00001	0.00001	0.99996	0.00001	0.00001
Norin_20	temperate_japonica	0.99996	0.00001	0.00001	0.99996	0.00001	0.00001
NOVARA	temperate_japonica	0.99996	0.00001	0.00001	0.99996	0.00001	0.00001
OLYMPIADA	indica	0.99996	0.99996	0.00001	0.00001	0.00001	0.00001
OPALE	temperate_japonica	0.99996	0.00001	0.00001	0.99996	0.00001	0.00001
ORIGINARIO	temperate_japonica	0.99996	0.00001	0.00001	0.99996	0.00001	0.00001
ORIONE	temperate_japonica	0.99996	0.00001	0.00001	0.99996	0.00001	0.00001
OSTIGLIA	temperate_japonica	0.99996	0.00001	0.00001	0.99996	0.00001	0.00001
OTA	temperate_japonica	0.99996	0.00001	0.00001	0.99996	0.00001	0.00001
P6	temperate_japonica	0.99996	0.00001	0.00001	0.99996	0.00001	0.00001
PADANO	temperate_japonica	0.99996	0.00001	0.00001	0.99996	0.00001	0.00001
PEGONIL	temperate_japonica	0.99996	0.00001	0.00001	0.99996	0.00001	0.00001
PELDE	temperate_japonica	0.99996	0.00001	0.00001	0.99996	0.00001	0.00001
PERLA	temperate_japonica	0.99996	0.00001	0.00001	0.99996	0.00001	0.00001
Phudugey	aus	0.99996	0.00001	0.00001	0.00001	0.00001	0.99996
PIEMONTE	temperate_japonica	0.99996	0.00001	0.00001	0.99996	0.00001	0.00001
PLOVDIV_22	temperate_japonica	0.99996	0.00001	0.00001	0.99996	0.00001	0.00001
PLOVDIV_24	temperate_japonica	0.99996	0.00001	0.00001	0.99996	0.00001	0.00001
PLUS	tropical_japonica	0.99996	0.00001	0.99996	0.00001	0.00001	0.00001
POLIZESTI_28	temperate_japonica	0.99996	0.00001	0.00001	0.99996	0.00001	0.00001
POSEIDONE	temperate_japonica	0.99996	0.00001	0.00001	0.99996	0.00001	0.00001
Pratao	tropical_japonica	0.99996	0.00001	0.99996	0.00001	0.00001	0.00001
PRECOCE_6	temperate_japonica	0.99996	0.00001	0.00001	0.99996	0.00001	0.00001
PRECOCE_ROSSI	temperate_japonica	0.99996	0.00001	0.00001	0.99996	0.00001	0.00001
PRECOZ_2FA	temperate_japonica	0.99996	0.00001	0.00001	0.99996	0.00001	0.00001
PROMETEO	temperate_japonica	0.99996	0.00001	0.00001	0.99996	0.00001	0.00001
PUNTAL	tropical_japonica	0.99996	0.00001	0.99996	0.00001	0.00001	0.00001
R_271	temperate_japonica	0.99996	0.00001	0.00001	0.99996	0.00001	0.00001
RADON	temperate_japonica	0.99996	0.00001	0.00001	0.99996	0.00001	0.00001
RANGHINO	temperate_japonica	0.99996	0.00001	0.00001	0.99996	0.00001	0.00001
Rathuwee	indica	0.99996	0.99996	0.00001	0.00001	0.00001	0.00001
RAZZA_77	temperate_japonica	0.99996	0.00001	0.00001	0.99996	0.00001	0.00001
RB_GAMMA	temperate_japonica	0.99996	0.00001	0.00001	0.99996	0.00001	0.00001
REXMONT	tropical_japonica	0.99996	0.00001	0.99996	0.00001	0.00001	0.00001
RIBE	temperate_japonica	0.99996	0.00001	0.00001	0.99996	0.00001	0.00001
RIBE_JAUNE	temperate_japonica	0.99996	0.00001	0.00001	0.99996	0.00001	0.00001
RINALDO_BERSANI	temperate_japonica	0.99996	0.00001	0.00001	0.99996	0.00001	0.00001
RINGO	temperate_japonica	0.99996	0.00001	0.00001	0.99996	0.00001	0.00001
ROBBIO_SEL1	temperate_japonica	0.99996	0.00001	0.00001	0.99996	0.00001	0.00001
RODEO	temperate_japonica	0.99996	0.00001	0.00001	0.99996	0.00001	0.00001

RODINA	temperate_japonica	0.99996	0.00001	0.00001	0.99996	0.00001	0.00001
ROMA	temperate_japonica	0.99996	0.00001	0.00001	0.99996	0.00001	0.00001
ROTUNDUS	temperate_japonica	0.99996	0.00001	0.00001	0.99996	0.00001	0.00001
ROXANI	temperate_japonica	0.99996	0.00001	0.00001	0.99996	0.00001	0.00001
RPC_12	temperate_japonica	0.99996	0.00001	0.00001	0.99996	0.00001	0.00001
RUSSO	temperate_japonica	0.99996	0.00001	0.00001	0.99996	0.00001	0.00001
S101	temperate_japonica	0.99996	0.00001	0.00001	0.99996	0.00001	0.00001
S102	temperate_japonica	0.99996	0.00001	0.00001	0.99996	0.00001	0.00001
S102_2	temperate_japonica	0.99996	0.00001	0.00001	0.99996	0.00001	0.00001
SAEDINENIE	temperate_japonica	0.99996	0.00001	0.00001	0.99996	0.00001	0.00001
SAFARI	temperate_japonica	0.99996	0.00001	0.00001	0.99996	0.00001	0.00001
SAKHA_102	temperate_japonica	0.99996	0.00001	0.00001	0.99996	0.00001	0.00001
SAKHA_103	temperate_japonica	0.99996	0.00001	0.00001	0.99996	0.00001	0.00001
SALOIO	temperate_japonica	0.99996	0.00001	0.00001	0.99996	0.00001	0.00001
SANDORA	temperate_japonica	0.99996	0.00001	0.00001	0.99996	0.00001	0.00001
SANT_ANDREA	temperate_japonica	0.99996	0.00001	0.00001	0.99996	0.00001	0.00001
SATURNO	tropical_japonica	0.99996	0.00001	0.99996	0.00001	0.00001	0.00001
SCUDO	tropical_japonica	0.99996	0.00001	0.99996	0.00001	0.00001	0.00001
SELENIO	temperate_japonica	0.99996	0.00001	0.00001	0.99996	0.00001	0.00001
SELN_244A620	temperate_japonica	0.99996	0.00001	0.00001	0.99996	0.00001	0.00001
SENIA	temperate_japonica	0.99996	0.00001	0.00001	0.99996	0.00001	0.00001
SEQUIAL	temperate_japonica	0.99996	0.00001	0.00001	0.99996	0.00001	0.00001
SESIAMOCHI	temperate_japonica	0.99996	0.00001	0.00001	0.99996	0.00001	0.00001
SETANTUNO	temperate_japonica	0.99996	0.00001	0.00001	0.99996	0.00001	0.00001
SHANGHAI	aus	0.99996	0.00001	0.00001	0.00001	0.00001	0.99996
Shinrike	temperate_japonica	0.99996	0.00001	0.00001	0.99996	0.00001	0.00001
Shoemed	temperate_japonica	0.99996	0.00001	0.00001	0.99996	0.00001	0.00001
SHSS_381	temperate_japonica	0.99996	0.00001	0.00001	0.99996	0.00001	0.00001
SHSS_53	temperate_japonica	0.99996	0.00001	0.00001	0.99996	0.00001	0.00001
SIRIO_CL	tropical_japonica	0.99996	0.00001	0.99996	0.00001	0.00001	0.00001
SIS_R215	tropical_japonica	0.99996	0.00001	0.99996	0.00001	0.00001	0.00001
SLAVA	temperate_japonica	0.99996	0.00001	0.00001	0.99996	0.00001	0.00001
SMERALDO	temperate_japonica	0.99996	0.00001	0.00001	0.99996	0.00001	0.00001
SP55	temperate_japonica	0.99996	0.00001	0.00001	0.99996	0.00001	0.00001
SPRINT	tropical_japonica	0.99996	0.00001	0.99996	0.00001	0.00001	0.00001
SR_113	temperate_japonica	0.99996	0.00001	0.00001	0.99996	0.00001	0.00001
Suweon_362	temperate_japonica	0.99996	0.00001	0.00001	0.99996	0.00001	0.00001
T_1	aus	0.99996	0.00001	0.00001	0.00001	0.00001	0.99996
T757	temperate_japonica	0.99996	0.00001	0.00001	0.99996	0.00001	0.00001
Taducan	indica	0.99996	0.99996	0.00001	0.00001	0.00001	0.00001
TAICHUNG_65	temperate_japonica	0.99996	0.00001	0.00001	0.99996	0.00001	0.00001
TEA	temperate_japonica	0.99996	0.00001	0.00001	0.99996	0.00001	0.00001
TEJO	temperate_japonica	0.99996	0.00001	0.00001	0.99996	0.00001	0.00001
TEQING	indica	0.99996	0.99996	0.00001	0.00001	0.00001	0.00001
TEXMONT	tropical_japonica	0.99996	0.00001	0.99996	0.00001	0.00001	0.00001
THAIBONNET	tropical_japonica	0.99996	0.00001	0.99996	0.00001	0.00001	0.00001
THAIPERLA	temperate_japonica	0.99996	0.00001	0.00001	0.99996	0.00001	0.00001
TIMICH_108	temperate_japonica	0.99996	0.00001	0.00001	0.99996	0.00001	0.00001
TITANIO	temperate_japonica	0.99996	0.00001	0.00001	0.99996	0.00001	0.00001
TITANO (ATLANTE)	tropical_japonica	0.99996	0.00001	0.99996	0.00001	0.00001	0.00001
TOPAZIO	temperate_japonica	0.99996	0.00001	0.00001	0.99996	0.00001	0.00001
TORIO	temperate_japonica	0.99996	0.00001	0.00001	0.99996	0.00001	0.00001
Trembese	tropical_japonica	0.99996	0.00001	0.99996	0.00001	0.00001	0.00001
ULISSE	temperate_japonica	0.99996	0.00001	0.00001	0.99996	0.00001	0.00001
ULLAL	temperate_japonica	0.99996	0.00001	0.00001	0.99996	0.00001	0.00001
UPLA_77	tropical_japonica	0.99996	0.00001	0.99996	0.00001	0.00001	0.00001
UPLA_79	tropical_japonica	0.99996	0.00001	0.99996	0.00001	0.00001	0.00001
UPLA_91	tropical_japonica	0.99996	0.00001	0.99996	0.00001	0.00001	0.00001
URANO	tropical_japonica	0.99996	0.00001	0.99996	0.00001	0.00001	0.00001
VALTEJO	temperate_japonica	0.99996	0.00001	0.00001	0.99996	0.00001	0.00001

VARIETA_16	temperate_japonica	0.99996	0.00001	0.00001	0.99996	0.00001	0.00001
VENERE	temperate_japonica	0.99996	0.00001	0.00001	0.99996	0.00001	0.00001
VIALE	temperate_japonica	0.99996	0.00001	0.00001	0.99996	0.00001	0.00001
VIALONE_190	temperate_japonica	0.99996	0.00001	0.00001	0.99996	0.00001	0.00001
VIALONE_NANO	temperate_japonica	0.99996	0.00001	0.00001	0.99996	0.00001	0.00001
VICTORIA	temperate_japonica	0.99996	0.00001	0.00001	0.99996	0.00001	0.00001
VIRGO	temperate_japonica	0.99996	0.00001	0.00001	0.99996	0.00001	0.00001
VOLANO	temperate_japonica	0.99996	0.00001	0.00001	0.99996	0.00001	0.00001
YRL_196	temperate_japonica	0.99996	0.00001	0.00001	0.99996	0.00001	0.00001
YRM_6_2	temperate_japonica	0.99996	0.00001	0.00001	0.99996	0.00001	0.00001
ZENITH	tropical_japonica	0.99996	0.00001	0.99996	0.00001	0.00001	0.00001
ZHENSHANG_97	indica	0.99996	0.99996	0.00001	0.00001	0.00001	0.00001
RUBINO	temperate_japonica	0.99996	0.00001	0.00001	0.99996	0.00001	0.00001
RONCOLO	temperate_japonica	0.99996	0.00001	0.00001	0.99996	0.00001	0.00001
OLCENENGO	temperate_japonica	0.99996	0.00001	0.00001	0.99996	0.00001	0.00001
SOURE	temperate_japonica	0.99996	0.00002	0.00001	0.99996	0.00001	0.00001
NIBBIO	temperate_japonica	0.99995	0.00002	0.00001	0.99995	0.00001	0.00001
REDI	temperate_japonica	0.99995	0.00001	0.00002	0.99995	0.00001	0.00001
VENERIA	temperate_japonica	0.99995	0.00002	0.00001	0.99995	0.00001	0.00001
BALDO	temperate_japonica	0.99538	0.00001	0.00459	0.99538	0.00001	0.00001
STRELLA	temperate_japonica	0.99356	0.00001	0.00641	0.99356	0.00001	0.00001
CERVO	temperate_japonica	0.98967	0.00001	0.01030	0.98967	0.00001	0.00001
ONDA	temperate_japonica	0.98727	0.00001	0.01270	0.98727	0.00001	0.00001
M6	temperate_japonica	0.97842	0.00001	0.02155	0.97842	0.00001	0.00001
ADELAIDE_CHIAPPELLI	temperate_japonica	0.97784	0.00001	0.02213	0.97784	0.00001	0.00001
SILLA	temperate_japonica	0.97184	0.00001	0.02813	0.97184	0.00001	0.00001
MOLO	temperate_japonica	0.97161	0.00001	0.02836	0.97161	0.00001	0.00001
CALENDAL	temperate_japonica	0.96708	0.00001	0.03289	0.96708	0.00001	0.00001
TOSCA	temperate_japonica	0.96355	0.00002	0.03641	0.96355	0.00001	0.00001
PIERINA_MARCHETTI	temperate_japonica	0.95146	0.00001	0.04851	0.95146	0.00001	0.00001
RIZZOTTO_51_1	temperate_japonica	0.94355	0.00001	0.05642	0.94355	0.00001	0.00001
SESIA	temperate_japonica	0.91730	0.00001	0.08267	0.91730	0.00001	0.00001
ALICE	temperate_japonica	0.91206	0.00001	0.08791	0.91206	0.00001	0.00001
MANTOVA	temperate_japonica	0.90435	0.00001	0.09562	0.90435	0.00001	0.00001
FAMILIA_181	temperate_japonica	0.89653	0.00001	0.10344	0.89653	0.00001	0.00001
FAISCA	temperate_japonica	0.88146	0.00001	0.10962	0.88146	0.00001	0.00890
BONNI	temperate_japonica	0.87482	0.00001	0.12515	0.87482	0.00001	0.00001
SUPER	temperate_japonica	0.86903	0.00001	0.13094	0.86903	0.00001	0.00001
EUROSE	temperate_japonica	0.86032	0.00001	0.13965	0.86032	0.00001	0.00001
GRITNA	temperate_japonica	0.85951	0.00001	0.14046	0.85951	0.00001	0.00001
BORRACHO	temperate_japonica	0.84453	0.00001	0.14934	0.84453	0.00001	0.00611
SAVIO	temperate_japonica	0.84177	0.00001	0.15820	0.84177	0.00001	0.00001
HAREM	temperate_japonica	0.84094	0.00001	0.15292	0.84094	0.00001	0.00612
ARTICO	indica	0.83992	0.83992	0.16005	0.00001	0.00001	0.00001
SENATORE_NOVELLI	temperate_japonica	0.83754	0.00001	0.16243	0.83754	0.00001	0.00001
RUBIDIO	temperate_japonica	0.82836	0.00001	0.17161	0.82836	0.00001	0.00001
RUBI	temperate_japonica	0.82734	0.00808	0.15675	0.82734	0.00001	0.00782
RIVA	temperate_japonica	0.80991	0.00001	0.19006	0.80991	0.00001	0.00001
LORD	temperate_japonica	0.80443	0.00001	0.19554	0.80443	0.00001	0.00001
ZENA	tropical_japonica	0.80305	0.00001	0.80305	0.19692	0.00001	0.00001
SANDOCA	temperate_japonica	0.80035	0.00519	0.18896	0.80035	0.00001	0.00548
GALILEO	temperate_japonica	0.78458	0.00001	0.21539	0.78458	0.00001	0.00001
CT36	temperate_japonica	0.77946	0.00001	0.22051	0.77946	0.00001	0.00001
ARTIGLIO	indica	0.76156	0.76156	0.23629	0.00001	0.00214	0.00001
GHIBLI	temperate_japonica	0.76098	0.00001	0.23899	0.76098	0.00001	0.00001
CRESO	temperate_japonica	0.76047	0.00001	0.23950	0.76047	0.00001	0.00001
UPLA_75	tropical_japonica	0.75430	0.01624	0.75430	0.22943	0.00001	0.00001
SCIROCCO	temperate_japonica	0.74414	0.00001	0.25583	0.74414	0.00001	0.00001
UPLA_32	tropical_japonica	0.74356	0.00001	0.74356	0.21946	0.03696	0.00001
UPLA_80	tropical_japonica	0.73384	0.00001	0.73384	0.26613	0.00001	0.00001

BASMATI_DETRADUNI	indica	0.72967	0.72967	0.00001	0.00001	0.27030	0.00001
BIANCA	temperate_japonica	0.71994	0.00001	0.28003	0.71994	0.00001	0.00001
VELA	temperate_japonica	0.70343	0.00002	0.29653	0.70343	0.00001	0.00001
MELAS	temperate_japonica	0.70329	0.00001	0.29668	0.70329	0.00001	0.00001
UPLA_63	tropical_japonica	0.69475	0.03397	0.69475	0.27126	0.00001	0.00001
TARRISO	tropical_japonica	0.68340	0.00001	0.68340	0.31657	0.00001	0.00001
UPLA_104	tropical_japonica	0.68254	0.00001	0.68254	0.31744	0.00001	0.00001
PANDA	tropical_japonica	0.68064	0.00001	0.68064	0.31933	0.00001	0.00001
SAGRES	temperate_japonica	0.67600	0.00356	0.30580	0.67600	0.00001	0.01463
UPLA_66	tropical_japonica	0.67470	0.08384	0.67470	0.24144	0.00001	0.00001
MIARA	temperate_japonica	0.66760	0.00002	0.33236	0.66760	0.00001	0.00001
UPLA_68	tropical_japonica	0.65017	0.12995	0.65017	0.21986	0.00001	0.00001
CARIOCA	tropical_japonica	0.63819	0.00001	0.63819	0.36178	0.00001	0.00001
SAMBA	tropical_japonica	0.63752	0.00001	0.63752	0.36246	0.00001	0.00001
GRAAL	tropical_japonica	0.63547	0.00001	0.63547	0.36450	0.00001	0.00001
CHACARERO	temperate_japonica	0.63525	0.00001	0.36472	0.63525	0.00001	0.00001
ALINANO	temperate_japonica	0.62619	0.04813	0.18637	0.62619	0.04017	0.09914
ELLEBI	tropical_japonica	0.62329	0.00001	0.62329	0.37668	0.00001	0.00001
NANO	temperate_japonica	0.61901	0.00001	0.38095	0.61901	0.00001	0.00001
CT23	temperate_japonica	0.61659	0.00001	0.38338	0.61659	0.00001	0.00001
MEJANES	temperate_japonica	0.61435	0.00001	0.38561	0.61435	0.00001	0.00001
GIANO	tropical_japonica	0.61367	0.02008	0.61367	0.36623	0.00001	0.00001
ITALPATNAxMILYANG	temperate_japonica	0.61312	0.29224	0.09463	0.61312	0.00001	0.00001
BAJANGxALLORIO	temperate_japonica	0.60607	0.00001	0.39390	0.60607	0.00001	0.00001
SALVO	tropical_japonica	0.59880	0.00002	0.59880	0.36583	0.03534	0.00001
DARDO	tropical_japonica	0.58469	0.00001	0.58469	0.41528	0.00001	0.00001
EUROSIS	temperate_japonica	0.57250	0.00001	0.42747	0.57250	0.00001	0.00001
SANTERNO	temperate_japonica	0.56696	0.00001	0.43301	0.56696	0.00001	0.00001
ALBATROS	temperate_japonica	0.56127	0.00001	0.43871	0.56127	0.00001	0.00001
GREPPI	tropical_japonica	0.55341	0.00001	0.55341	0.44656	0.00001	0.00001
ESTRELA	temperate_japonica	0.54767	0.04722	0.34711	0.54767	0.00244	0.05556
UPLA_64	tropical_japonica	0.53701	0.11984	0.53701	0.34313	0.00001	0.00001
MAIORAL	temperate_japonica	0.53691	0.00001	0.44510	0.53691	0.00001	0.01797
PREVER	tropical_japonica	0.53110	0.00001	0.53110	0.46887	0.00001	0.00001
ERMES	tropical_japonica	0.53002	0.00001	0.53002	0.45102	0.01893	0.00001
PECOS	tropical_japonica	0.52785	0.00001	0.52785	0.47212	0.00001	0.00001
ANSEATICO	temperate_japonica	0.52538	0.00001	0.47459	0.52538	0.00001	0.00001
ARTEMIDE	tropical_japonica	0.51416	0.00001	0.51416	0.46085	0.00906	0.01592
BENGAL	temperate_japonica	0.50951	0.01473	0.47575	0.50951	0.00001	0.00001
MIDA	temperate_japonica	0.50621	0.00001	0.49377	0.50621	0.00001	0.00001
GRALDO	tropical_japonica	0.50375	0.00001	0.50375	0.49622	0.00001	0.00001
RONALDO	tropical_japonica	0.50135	0.00001	0.50135	0.49862	0.00001	0.00001
ROMBO	temperate_japonica	0.49691	0.38250	0.12057	0.49691	0.00001	0.00001
OSCARxSUWEON	temperate_japonica	0.49230	0.38791	0.11298	0.49230	0.00680	0.00001
FIDJI	tropical_japonica	0.40973	0.04323	0.40973	0.22448	0.32255	0.00001
