# Supplementary material for: Genome-Wide Association Study for Traits Related to Plant and Grain Morphology, and Root Architecture in Temperate Rice Accessions
Source: PLoS One. 2016 May 26;11(5):e0155425. doi: 10.1371/journal.pone.0155425 (PMC4881974; doi:10.1371/journal.pone.0155425)
Supplement: S4 Table — Associations between SNP genotypes and plant height in the region of the gene SD1 on chromosome 1, from a linear GWAS model without accounting for population structure. (PDF) [file pone.0155425.s006.pdf]

Genome-wide association study for traits related to plant and grain morphology, and root architecture in temperate rice accessions

Filippo Biscarini[1,*] et al.
1 Department of Bioinformatics and Biostatistics, PTP Science Park, Lodi, Italy
$*$ E-mail: filippo.biscarini@ptp.it

Table 1: **Associations between SNP genotypes and plant height in the region of the gene SD1 on chromosome 1, from a linear GWAS model without accounting for population structure**

Trait	Chr	Marker	Position	p.value	Candidate.Gene
PH	1	S1_330484	330.484	7.2100000000E-05	
PH	1	S1_23553310	23553.31	3.0400000000E-05	
PH	1	S1_30310666	30310.666	8.9500000000E-05	
PH	1	S1_35155442	35155.442	2.6600000000E-05	
PH	1	S1_35586856	35586.856	8.1900000000E-06	
PH	1	S1_35847644	35847.644	4.7000000000E-06	
PH	1	S1_36682877	36682.877	4.5300000000E-06	
PH	1	S1_37012118	37012.118	4.0500000000E-06	
PH	1	S1_37403082	37403.082	8.2200000000E-06	
PH	1	S1_38197794	38197.794	5.8400000000E-10	
PH	1	S1_38457643	38457.643	1.7700000000E-10	SD1 (Sasaki et al. 2002)
PH	1	S1_38718425	38718.425	2.4000000000E-10	
PH	1	S1_39286700	39286.7	7.1600000000E-09	
PH	1	S1_39711918	39711.918	1.2500000000E-07	
PH	1	S1_40254090	40254.09	2.0300000000E-07	
PH	1	S1_40964141	40964.141	6.8800000000E-08	
PH	1	S1_41298835	41298.835	1.4200000000E-07	
PH	1	S1_42690322	42690.322	3.5800000000E-06	
PH	1	S1_43143774	43143.774	4.2300000000E-06	
